# Multi-tissue multi-omics integration reveals tissue-specific pathways, gene networks and drug candidates for type 1 diabetes

**DOI:** 10.1007/s00125-026-06721-6

**Published:** 2026-04-15

**Authors:** Montgomery Blencowe, Zara Saleem, Ruoshui Liu, Margaret Wang, I-Hsin Tseng, Julian Wier, Stefan Mutter, Florin Vaida, Yi Guo, Niina Sandholm, Courtney Ackeifi, Daniel L. Kaufman, Xia Yang

**Affiliations:** 1 Department of Integrative Biology and Physiology, University of California, Los Angeles, CA USA; 2 Interdepartmental Program of Molecular, Cellular and Integrative Physiology, University of California, Los Angeles, CA USA; 3 Interdepartmental Program of Bioinformatics – Systems Biology, University of California, Los Angeles, CA USA; 4 Research Program for Clinical and Molecular Metabolism, Faculty of Medicine, University of Helsinki, Helsinki, Finland; 5 Folkhälsan Research Center, Helsinki, Finland; 6 Department of Nephrology, University of Helsinki and Helsinki University Hospital, Helsinki, Finland; 7 Division of Biostatistics and Bioinformatics, School of Public Health, University of California San Diego, La Jolla, CA USA; 8 College of Medicine, University of Florida, Gainesville, FL USA; 9 Breakthrough T1D, New York, NY USA; 10 Department of Molecular and Medical Pharmacology, UCLA School of Medicine, University of California, Los Angeles, CA USA

**Keywords:** Drug repositioning, Electronic medical records, Gene networks, Genome-wide association studies, Key drivers, Multi-omics, Type 1 diabetes

## Abstract

**Aims/hypothesis:**

Although genome-wide association studies (GWAS) have identified loci associated with type 1 diabetes, the specific pathways and regulatory networks linking these loci to disease pathology remain largely unknown. We hypothesised that type 1 diabetes genetic risk factors disrupt tissue-specific biological pathways and gene networks that ultimately lead to beta cell loss.

**Methods:**

We conducted a multi-tissue multi-omics analysis that integrates human GWAS data for type 1 diabetes with tissue-specific regulatory data for gene expression and gene network models across relevant tissues to highlight key pathways and key driver (KD) genes contributing to type 1 diabetes pathogenesis. KD genes were validated using islet-specific gene expression and protein data from non-obese diabetic (NOD) mice compared with type 2 diabetic and non-diabetic mouse models. Drug repositioning predictions were generated using the INCS L1000 and PharmOmics platforms, and candidate drugs were tested using electronic medical records (EMRs) of individuals with type 1 diabetes from the OneFlorida+ Clinical Data Network.

**Results:**

Our integrative genomics approach identified known immune pathways across multiple tissues, such as adaptive immune responses, cytokine-mediated inflammation, primary immunodeficiency, and interactions between lymphoid and non-lymphoid cells. Tissue-specific signals included genes related to type 2 diabetes in lymphocytes, viral response pathways in macrophages and monocytes, and Notch signalling in adipose tissue and immune cells. In pancreatic islet analysis, we observed significant enrichment for type 1 diabetes and type 2 diabetes gene sets alongside immune-related pathways, including antigen processing, systemic lupus erythematosus and IFN signalling. Removing HLA genes from the analysis revealed additional immune pathways, such as retinoic acid-inducible gene I (RIG-I)/melanoma differentiation-associated protein 5 (MDA5) induction of interferons, together with melanogenesis, steroid hormone synthesis and iron transport. Network modelling highlighted the autoimmune basis of disease, with KDs such as FYN, TAP1, WAS and HLA-B/C/G, as well as additional immunomodulatory proteins such as LCK, LCP2 and genes such as *EMR1* and *GC*. These KDs were further supported by gene and protein expression data from NOD mice. We additionally highlight various drug classes that target the type 1 diabetes genetic networks and may be useful to delay type 1 diabetes development; some of these were supported by our EMR screen.

**Conclusions/interpretation:**

Our multi-tissue multi-omics approach provides a detailed landscape of the tissue-specific genetic networks and regulators underlying type 1 diabetes. This analysis confirms the roles of known immune pathways while uncovering additional regulatory elements and disease-associated networks, thus expanding our understanding of type 1 diabetes pathogenesis. The identification of potential drug candidates through network analysis, with supporting evidence from EMRs, offers potential therapeutic strategies for targeting disease pathways and holds promise for delaying or preventing type 1 diabetes progression.

**Graphical Abstract:**

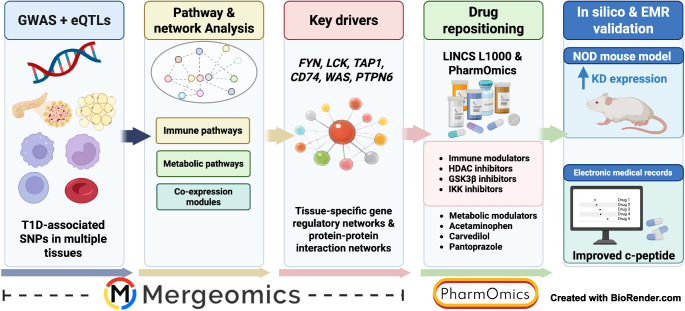

**Supplementary Information:**

The online version contains peer-reviewed but unedited supplementary material available at 10.1007/s00125-026-06721-6.


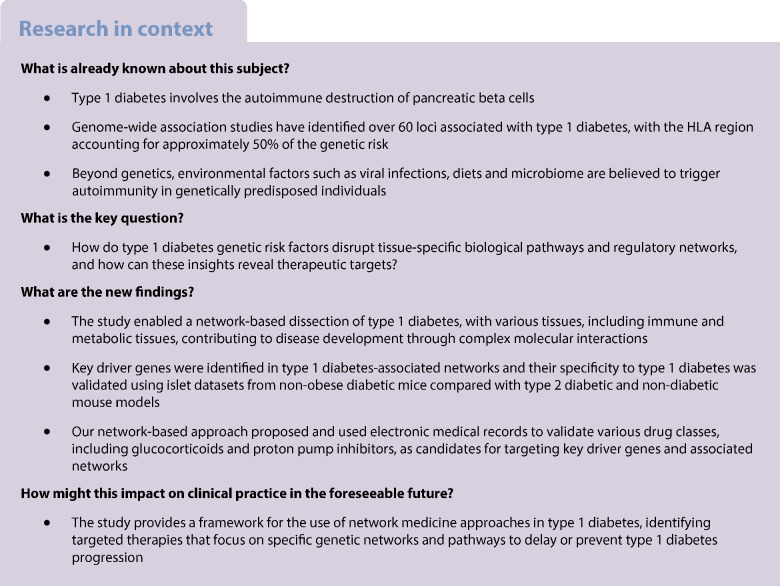


## Introduction

Type 1 diabetes is characterised as the autoimmune loss of pancreatic beta cells and resultant impairment of glucose homeostasis. Currently, type 1 diabetes affects approximately 8.5 million people globally, representing 5–10% of the total diabetic population, with an annual incidence increase of 2–3% [1–3]. The risk of developing type 1 diabetes is increased by approximately 5.6% or 50%, respectively, for individuals with a parent or monozygotic twin who has the disease, when compared with the general population. Parental heritability estimates predict that diabetic fathers confer an increased risk of type 1 diabetes development of approximately 12%, while that for diabetic mothers is approximately 6% [4]. Thus, there is a genetic component that predisposes an individual to type 1 diabetes. However, type 1 diabetes is not usually present in individuals with a family history, with only approximately 10% of individuals with type 1 diabetes having a first- or second-degree relative with the disease, thus also suggesting a significant environmental contribution [5]. Interestingly, there seems to be an increased disposition for developing type 1 diabetes for individuals who live in regions of Northern Europe, independent of genetic background [6, 7]. This environmental contribution has several potential manifestations, such as alterations in gut microbiota [8] or pre- and postnatal dietary factors, including early exposure to gluten [9] and vitamin D deficiency [10]. Furthermore, a longstanding hypothesis predicts that exposure to viral infection may also be a causal factor [11], particularly exposure to enteroviruses [12], which appear to target pancreatic islet cells [13]. Therefore, both genetic and environmental components contribute to type 1 diabetes incidence and progression, but there is a large gap in understanding the complex genetic and environmental architectures as well as the interaction between the two. It is plausible that genetic risks represent ‘first hits’ and environmental factors act as ‘second hits’, interacting with genetic predisposition to trigger type 1 diabetes development [14–16].

On the genetic front, genome-wide association studies (GWAS) have uncovered over 60 type 1 diabetes genetic risk loci. The main genes that predispose an individual to type 1 diabetes are located within the HLA region on chromosome 6, encoding the major histocompatibility complex (MHC), which is critical for adaptive immunity. While HLA-encoding genes have the strongest association and account for up to 50% of the total genetic type 1 diabetes risk [17, 18], loci outside the HLA region, including genes encoding protein tyrosine phosphatase, non-receptor type 22 (*PTPN22*), IL-2 receptor alpha (*IL2RA*), the insulin gene (*INS*) and cytotoxic T-lymphocyte-associated protein 4 (*CTLA4*), have also been shown to be associated with disease development [19]. However, these top loci at genome-wide significance cannot fully explain the total genetic heritability of type 1 diabetes. Moreover, the dominance of the HLA effect may overshadow additional unknown and important processes contributing to type 1 diabetes pathogenesis, with or without interactions with environmental factors. Identifying the missing genetic risks, or the ‘dark matter’, is important to gain a full understanding of the genetic underpinnings of type 1 diabetes pathogenesis. In addition, growing lines of evidence support an ‘omnigenic’ disease model [20], which states that a large proportion of genes in the genome may contribute to disease development through gene–gene interactions in networks within and between tissues, and key network regulators probably play more central roles than other peripheral disease-associated genes in the networks. In support of this, top GWAS loci for complex diseases have been found to be more concentrated in the periphery of gene networks and are less likely to be network regulators [21–26]. Therefore, simply focusing on the top GWAS hits will probably miss crucial regulatory genes. We hypothesised that type 1 diabetes genetic risks with a wide spectrum of effect size interact and perturb tissue-specific gene networks through a select set of regulatory genes, resulting in variations in type 1 diabetes susceptibility.

**Fig. 1:**
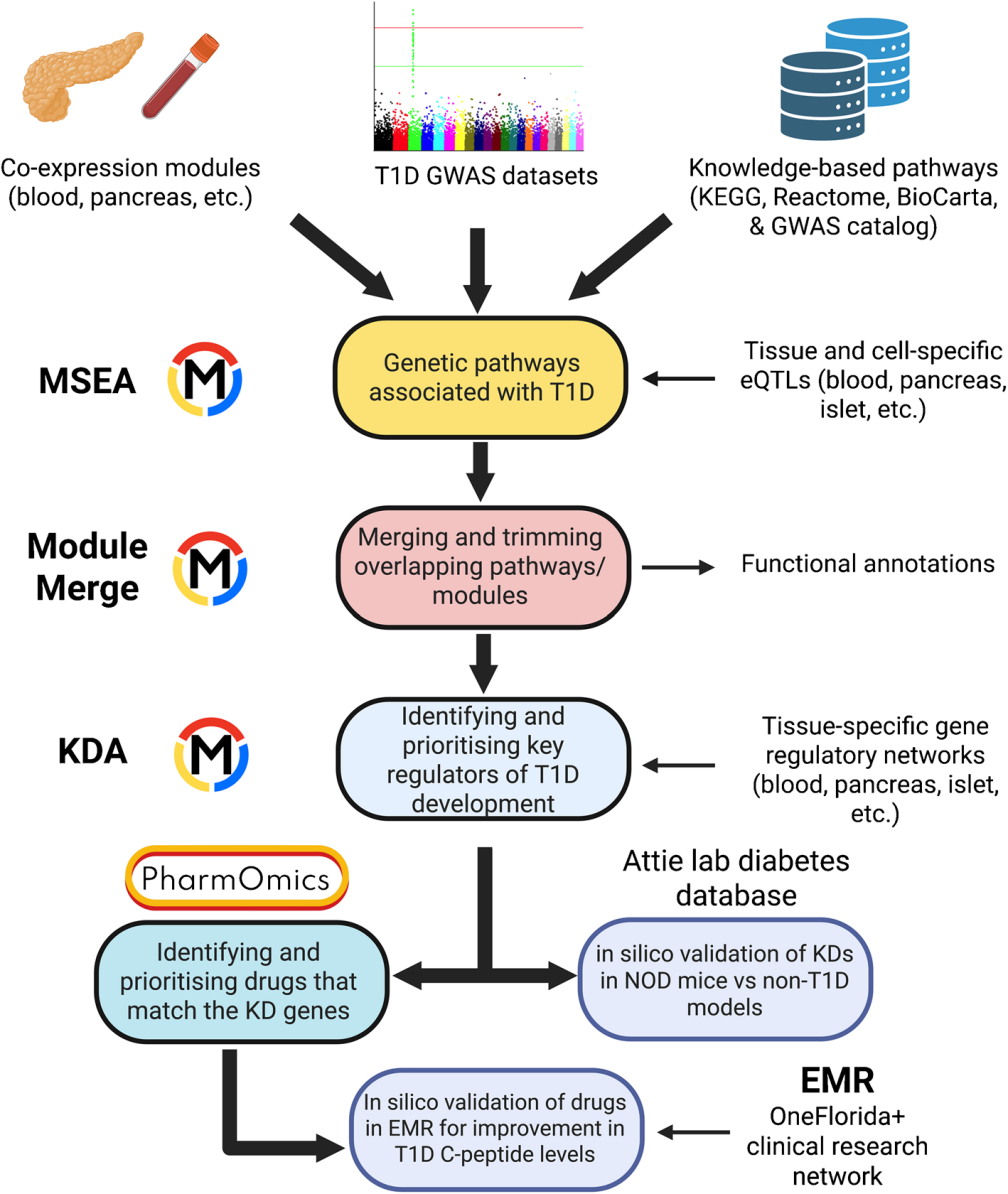
Overview of the study. We integrated diverse datasets using the Mergeomics framework to identify and prioritise therapeutic targets and drugs for type 1 diabetes (T1D). We collected and processed type 1 diabetes GWAS SNPs, mapping them to genes using tissue- and cell-specific eQTLs. The mapped genes were linked to canonical pathways (KEGG, Reactome, BioCarta and GWAS Catalog) and tissue-specific co-expression modules, with overlapping pathways and modules merged and refined to reduce redundancy. We then implemented KDA using tissue-specific GRNs, including PPI and Bayesian networks, to identify KD genes. These KDs were validated in silico using the Attie Lab Diabetes Database by comparing NOD mice with mouse models of type 2 diabetes and without diabetes. Drug repositioning was performed using PharmOmics to identify drugs targeting the KD genes, and candidate drugs were further evaluated in silico using EMRs from the OneFlorida+ Clinical Research Network for evidence of improvement in C-peptide levels in individuals with type 1 diabetes

Integration of GWAS data with functional genomics information such as tissue-specific expression quantitative trait loci (eQTLs) and gene networks has proven to be a powerful tool to pinpoint genes and their associated pathogenic mechanisms and regulators within the context of specific tissues [21–24, 27–29]. In this study, we use a computational pipeline, Mergeomics (Fig. 1) [23, 29] to integrate type 1 diabetes GWAS with tissue-specific functional information, such as the genetics of gene expression and gene regulatory networks (GRNs), from a broad range of type 1 diabetes relevant tissues or cell types [30–39]. By understanding how type 1 diabetes genetic risks affect gene networks within and across tissues, and by identifying key regulators, our study offers comprehensive insights into type 1 diabetes pathogenesis, and helps to prioritise regulators and potential therapeutic strategies.

## Methods

### Overview of study design

As shown in Fig. 1, we first assessed which biological pathways were enriched for type 1 diabetes GWAS signals [40] between type 1 diabetes and control individuals from two independent cohorts (see below). We used the full GWAS summary statistics for single nucleotide polymorphisms (SNPs) filtered by *r*^2^ >0.7 (see electronic supplementary material [ESM] Methods).

To identify tissue-specific pathways or gene networks associated with type 1 diabetes GWAS SNPs, we used tissue-specific eQTLs to guide SNP-to-gene mapping for each tissue. This analysis included a total of 18 eQTL sets from the GTEx database [41], the Cardiogenics Consortium [42] and the DICE study [43], and pancreatic islet-specific eQTLs [5]. The genes mapped to the GWAS SNPs through each tissue-specific eQTL set were analysed against biological pathways based on literature-based functional categories and tissue-specific gene co-expression network modules that define data-driven functional gene sets, using marker set enrichment analysis (MSEA) from Mergeomics (XiaYangLabOrg GitHub repository: https://github.com/XiaYangLabOrg/mergeomics, accessed 01 March 2025) [23, 29]. This approach identifies functionally related gene sets based on gene expression patterns, and has previously helped to derive novel biological insights into various diseases [21–26]. Integration of type 1 diabetes GWAS, tissue-specific eQTLs, and pathways and co-expression modules using MSEA reveals pathways and modules that are enriched for stronger genetic associations with type 1 diabetes compared with random gene sets. To prioritise the tissues on which to focus the downstream analysis, we used the tissues that had the highest number of significantly enriched pathways (false discovery rate [FDR] <5%), as these tissues probably play a more important role in type 1 diabetes pathogenesis, as informed by the GWAS and eQTL data.

After the MSEA, we carried out a meta-MSEA to meta-analyse the two independent GWAS datasets to look for shared pathways/modules, which we further simplified into independent ‘supersets’ to reduce redundancy between similar pathways/modules. To identify regulatory genes of these supersets, we mapped them to GRNs and protein–protein interaction (PPI) networks to identify key drivers (KDs) using key driver analysis (KDA). KDs are central network genes whose network neighbours are highly enriched for genes in the type 1 diabetes pathways/modules. We further carried out in silico validation using curated islet gene expression and protein data from eight strains of mice to highlight whether our KDs showed significantly higher/lower expression at the mRNA and/or protein level in non-obese diabetic (NOD) mice, a spontaneous autoimmune type 1 diabetes model, compared with control strains.

Lastly, we used our KDs as input into the LINCS L1000 perturbational gene expression database and PharmOmics to highlight potential drug candidates for type 1 diabetes treatment [44, 45]. Drugs were prioritised based on matching of type 1 diabetes KDs across multiple tissues with drug signatures, and predicted drugs were further validated by assessing changes in C-peptide levels in individuals with type 1 diabetes using electronic medical records (EMRs) from the OneFlorida+ Clinical Data Network (see below).

No ethics permission is required for this study.

### Type 1 diabetes GWAS datasets

The summary statistics of GWAS for type 1 diabetes were obtained from the JDRF/Wellcome Diabetes and Inflammation Laboratory, University of Oxford, UK [40, 46]. The study comprised 5913 type 1 diabetes individuals of European descent [38, 46], comprising 3983 from the UK GRID who were genotyped using the Illumina HumanHap550 version 3 (550k) Infinium Beadchip, and 1930 from the WTCCC who were genotyped using Affymetrix 500K. There were a total of 8828 control individuals, comprising 3999 from the 1958 birth cohort who were genotyped using the Illumina HumanHap550 version 3 (550k) Infinium Beadchip, 1490 who were genotyped using Affymetrix 500K (1958 birth cohort), 1455 from the UK Blood Services (UKBS) who were genotyped using Affymetrix 500K, and 1884 from a cohort of people with bipolar disorders who were genotyped using Affymetrix 500K [38, 46].

The above type 1 diabetes and control individuals were partitioned into two independent cohorts based on matching genotyping platforms: cohort 1 (Affymetrix) comprised 1930 individuals with type 1 diabetes and 4829 control individuals; cohort 2 (Illumina) comprised 3983 individuals with type 1 diabetes and 3999 control individuals (see ESM Methods).

### Mapping SNPs to genes

To link GWAS SNPs to their potential target genes, tissue-specific eQTLs were used to provide functional insight for the role of SNPs in gene expression regulation within a given tissue. Thirteen eQTL sets were obtained from the GTEx database (subcutaneous adipose tissue, visceral omentum adipose tissue, blood, brain, colon, heart, liver, lymphocyte, muscle, pancreas, pituitary, spleen and stomach) [47]. Additionally, we obtained macrophage and monocyte eQTLs from the Cardiogenics Consortium [40], pancreatic islet-specific eQTLs from various sources [5], and immune cell eQTLs including lymphocytes from the DICE study [43]. In addition, we incorporated beta cell eQTLs from the InsPIRE Consortium [48] at FDR <5% to increase cell-type resolution within the islet. We additionally queried for type 1 diabetes-specific islet eQTLs; however, type 1 diabetes-stratified datasets were either unavailable or underpowered [49]. A broader spectrum of tissues was considered at this step to help objectively infer which tissues may be more informative for type 1 diabetes association. GWAS results containing summary statistics for SNP to disease associations were mapped to each tissue eQTL set separately to derive individual SNP–gene mapping sets reflecting tissue origins to allow assessment of tissue-specific signals.

A high degree of linkage disequilibrium (LD) was observed in the eQTL data, which may cause biases in the downstream analysis. For this reason, we removed redundant SNPs that had LD of *r*^2^ >0.7 with a chosen SNP (details in ESM Methods).

### Data-driven modules of co-expressed genes

In order to assess whether type 1 diabetes GWAS signals are enriched in specific gene subnetworks, we derived gene co-expression networks using tissue-specific transcriptomic datasets from the GTEx portal, including subcutaneous adipose tissue, visceral omentum adipose tissue, blood and pancreas datasets [23, 26, 39]. These tissues were chosen because of their relevance to type 1 diabetes [28, 29, 31]. The WGCNA package was used to construct co-expression networks based on gene expression profiles [50]. Each tissue network contains multiple ‘modules’, and each module comprises tens to hundreds or thousands of genes that show co-expression. A total of 272 co-expression modules were curated.

### Knowledge-based biological pathways

We used a total of 1827 canonical pathways from Reactome (version 45) [22], the BioCarta database [51] and the Kyoto Encyclopedia of Genes and Genomes (KEGG) database [52]. In addition to the knowledge-based pathways, we constructed a type 1 diabetes positive control gene set based on candidate causal genes curated in the GWAS Catalog (*p*<5.0 × 10^−8^) [53]. Similar control gene sets for coronary heart disease, type 2 diabetes and height were also constructed to compare with the type 1 diabetes positive control set. Non-HLA pathways included all 1827 canonical pathways with HLA genes removed.

### Marker set enrichment analysis

To identify co-expression modules and pathways that show evidence for genetic association with type 1 diabetes, we applied MSEA within the Mergeomics package [23, 26] to each of the GWAS cohorts separately in conjunction with the eQTL sources. MSEA employs a χ^2^-like statistic with multiple quantile thresholds to assess whether a co-expression module or pathway shows enrichment of functional disease SNPs (i.e. those that are likely to regulate gene expression as captured in eQTLs) compared with random chance. The Benjamini–Hochberg FDR was estimated across all co-expression modules and pathways tested for each GWAS. Gene sets were statistically significant if FDR <5% in at least one SNP–gene mapping set. To evaluate gene sets across both GWAS studies, we performed a meta-analysis at the module/pathway level using the meta-MSEA function in Mergeomics, to retrieve robust gene sets across both cohorts. Merging of overlapping pathways/co-expression modules into supersets was performed to reduce redundancy (see ESM Methods).

### Tissue-specific GRNs and KDA

Tissue-specific Bayesian GRNs of adipose tissue, blood, islet and pancreas tissue were previously constructed [23, 26], and PPI networks were obtained from the STRING database [54]. We additionally constructed a beta cell GRN using RIMBANet [55] based on beta cell-enriched expression data, integrating transcription factor priors and beta cell cis-eQTLs. We chose to focus on these tissue networks due to our MSEA results showing the strongest statistical significance for these tissues. Using these networks, we performed a KDA in Mergeomics to identify potential KDs whose network neighbours are enriched for genes within the type 1 diabetes-associated supersets uncovered by MSEA. The algorithm employed a χ^2^ -like statistic similar to that described for MSEA, and an FDR <5% was used to focus on top robust KDs.

### In silico validation of KDs

To determine the relevance of the KDs to type 1 diabetes, we examined the islet RNA sequencing and proteomics profiles from the Attie Lab Diabetes Database (http://diabetes.wisc.edu) [56–59] generated from eight genetically diverse Collaborative Cross founder mouse strains, including NOD/ShiLtJ (NOD), a mouse model that spontaneously develops autoimmune diabetes; NZO/HlLtJ (NZO), a type 2 diabetic/obesity-associated diabetic strain; and A/J, 129S1/SvImJ (129), C57BL/6J (B6), CAST/EiJ (CAST), PWK/PhJ (PWK) and WSB/EiJ (WSB), which are non-diabetic strains. Starting at 4 weeks of age, the mice were placed on a Western-style diet high in fat and sucrose for 16 weeks [56–59]. Most strains were euthanised at 20–22 weeks, except for NZO male mice, which were euthanised at 14 weeks. All groups remained euglycaemic except the NZO male mice, which were hyperglycaemic. We used Student’s *t* test on the RNA and protein expression levels for prioritised KDs between the NOD mice and B6 mice to evaluate significant differences.

We also incorporated data from a previously published study [60] that compared pancreatic islet gene expression profiles between prediabetic 2–3-week-old NOD mice and non-obese resistant (NOR) mice using a microarray. NOR mice serve as a diabetes-free, MHC II-matched control strain for NOD mice [61]. A total of 213 differentially expressed genes (DEGs) between NOD and NOR islets were obtained from this study and cross-referenced with the islet PPI network KDs and their neighbours to assess overlap.

### Drug repositioning

We used the online web tool LINCS L1000 to match compounds and drugs with our type 1 diabetes KDs of individual tissues [45]. We collated all compounds and drugs that matched with each KD list at a median tau score of ≥90 on the LINCS L1000 database, and then calculated the absolute mean for the median tau scores across blood (which includes macrophages and monocytes), lymphocytes, pancreas, pancreatic islets, and non-HLA analysis to identify the drugs/compounds whose gene signatures best matched each of our KD networks.

We also used PharmOmics for drug repurposing, focusing on the KDs from islets, adipose tissue, blood, lymphocytes, macrophages, monocytes and pancreas [44]. Drugs with gene signatures matching the KDs were filtered by significance (*p*<0.05), and the top 100 drugs for each tissue were selected.

### In silico drug validation

As the directionality of drug effects relative to disease signatures was not inferred directly, subsequent validation was pursued using EMRs from the OneFlorida+ Clinical Data Network. Individuals with type 1 diabetes aged 18 years and over were identified using ICD-10 code E10 and ICD-9 codes 250.01 and 250.03 [62]. The cohort included 72,274 individuals with type 1 diabetes, of whom 55.71% were female and 44.29% were male, with a mean birth year of 1967 (SD=17.3 years; median 1965). The racial composition of the cohort was 55.84% White, 29.85% Black or African American, 1.35% Asian, 0.21% American Indian or Alaska Native, 0.05% Native Hawaiian or other Pacific Islander, and 0.26% multiracial, with 2.26% classified as other, 0.29% refusing to answer, 0.14% with no information, and 9.10% unknown. Our analysis focused on C-peptide measurements (nmol/l). For each drug identified in the drug repositioning analysis, we determined the treatment duration based on the earliest recorded start date and the latest end date, and only included drugs taken for at least 30 days. We then calculated the mean C-peptide levels before and after drug use, with ‘before’ defined as 6 months, 1 year, or 2 years prior to treatment initiation, and ‘after’ defined as the corresponding periods following treatment cessation. A one-sided paired *t* test and corresponding confidence interval were used to identify drugs that significantly increased C-peptide levels (*p*<0.05) in individuals with type 1 diabetes. A minimum of five individuals per drug was required for analysis.

## Results

### Identification of consistent and divergent canonical pathways and gene co-expression modules associated with type 1 diabetes across cohorts and tissues

We first assessed which knowledge-based biological (canonical) pathways and data-driven gene co-expression modules were enriched for type 1 diabetes GWAS signals. The use of tissue-specific eQTLs served to guide SNP-to-gene mapping and to capture tissue-specific results. From the 18 eQTL sets used, we prioritised seven tissues as the most informative in terms of how many pathways/modules showed significant enrichment for type 1 diabetes GWAS signals at FDR <5% based on MSEA, and their biological relevance to type 1 diabetes: blood, lymphocytes, macrophages, monocytes, pancreas, subcutaneous adipose tissue and visceral omentum adipose tissue. We therefore focus on reporting the results from these seven informative tissues only, with results for other tissues serving to supplement the main results.

**Fig. 2:**
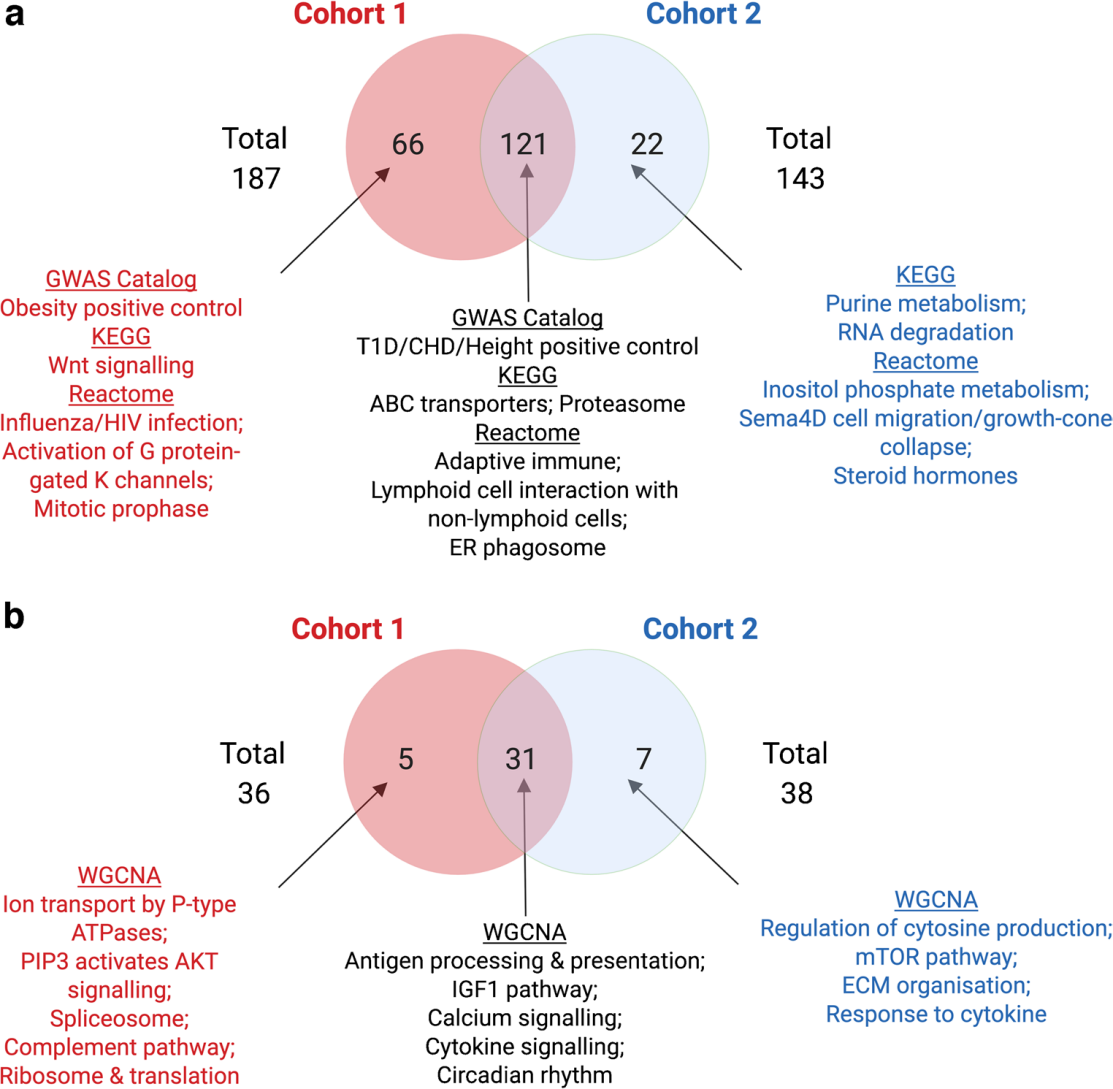
Enriched canonical pathways and co-expression modules for both type 1 diabetes GWAS cohorts. (**a**) The independent and overlapping knowledge-driven biological pathways (derived from KEGG, BioCarta, GWAS Catalog and Reactome) for both cohorts (FDR <5%). (**b**) Independent and overlapping data-driven co-expression modules (derived from WGCNA) for both cohorts (FDR <5%). T1D, type 1 diabetes

Out of the 1827 curated canonical pathways, we identified 187 pathways from cohort 1 enriched for type 1 diabetes GWAS association at an FDR <5% in at least one of the seven chosen tissues (ESM Table 1). From cohort 2, we found a total of 143 pathways enriched for type 1 diabetes GWAS association at an FDR <5% (ESM Table 2). Between cohort 1 and cohort 2, 121 pathways were significant in both (overlap fold enrichment =8.27, Fisher’s exact test *p*=5.84 × 10^−116^), 66 were unique to cohort 1, and 22 were unique to cohort 2 (Fig. 2a). The significant overlap in the enriched pathways across cohorts suggests reproducibility. We then used meta-MSEA to confirm significance across datasets (Fig. 2a). The shared pathways were mainly related to immune processes and the type 1 diabetes positive control gene set containing top type 1 diabetes hits from the GWAS Catalog (ESM Table 3). Unique pathways for cohort 1 included influenza/HIV infection, nuclear envelope breakdown and transport of mature mRNA. Unique pathways for cohort 2 included purine metabolism, inositol phosphate metabolism and RNA degradation.

We generated 272 data-driven gene co-expression modules from individual tissues from the GTEx transcriptomic database [39] using WGCNA [50], with which we integrated the cohort 1 and cohort 2 type 1 diabetes GWAS datasets. Cohort 1 showed enrichment in a total of 36 unique modules (FDR <5%) in at least one tissue (ESM Table 4), while cohort 2 showed significance in 38 modules (FDR <5%) (ESM Table 5). We found 31 modules that overlapped between the two cohorts (overlap fold enrichment =6.16, Fisher’s exact test *p*=6.76 × 10^−29^), including those associated with immune pathways, such as interferon signalling and cytokine signalling. We also identified five unique modules from cohort 1 (e.g. Akt signalling, complement pathway and ion transport) and seven unique modules from cohort 2 (e.g. cytosine production/response, mTOR pathways and extracellular matrix organisation) (Fig. 2b).

To assess the reproducibility of pathway enrichment within tissues, we compared the pathways identified in each tissue across cohort 1 and cohort 2 (ESM Fig. 1). All tissues demonstrated statistically significant overlap in the identified pathways between cohorts (*p*<0.05 by Fisher’s exact test), again supporting reproducibility.

We further assessed tissue specificity vs systemic changes in the significant canonical pathways and co-expression modules in both cohorts (ESM Table 6 for cohort 1 and ESM Table 7 for cohort 2). We found that monocytes, lymphocytes and blood contributed distinct processes. In contrast, macrophages, subcutaneous adipose tissue, visceral adipose tissue and pancreas were dominated by shared pathways. In addition, we found largely confirmatory results with the other tissue types tested across both cohorts (ESM Table 8 for cohort 1 and ESM Table 9 for cohort 2).

### Merging of pathways and co-expression modules into independent supersets

**Fig. 3:**
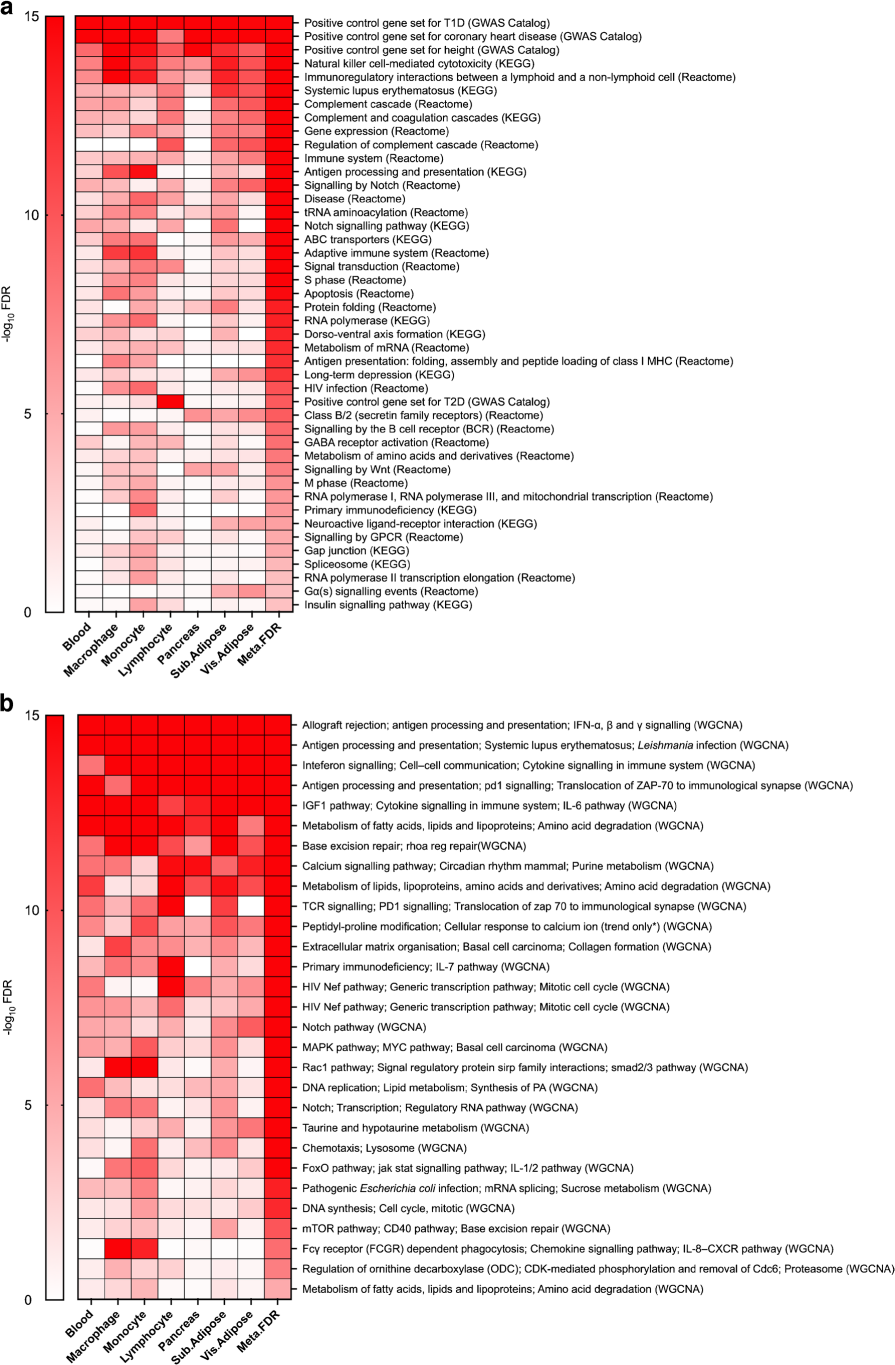
Tissue-specific meta-MSEA results from the combined cohort 1 and cohort 2 datasets for the supersets derived from the canonical pathways and co-expression network modules. (**a**) Heatmap for the statistical significance of type 1 diabetes genetic association across the supersets derived from the canonical pathways (KEGG, BioCarta and Reactome) (FDR <5%) in tissue-specific and cross-tissue analyses. (**b**) Heatmap for the statistical significance of type 1 diabetes genetic association across the superset-derived co-expression modules (WGCNA) (FDR <5%) in tissue-specific and cross-tissue analyses. ‘Trend only’ signifies that the given WGCNA module label did not pass an FDR<0.05 but was labelled with the most highly ranked pathway label match. Sub., subcutaneous; Vis., visceral; T1D, type 1 diabetes; T2D, type 2 diabetes

We focused on the 152 shared significant gene sets uncovered in our meta-analysis between cohort 1 and cohort 2 (31 co-expression modules and 121 canonical pathways) as they reflect reproducible signals for type 1 diabetes genetic association (Fig. 2a, b). As the co-expression modules and pathways were obtained from various sources, the gene sets had the potential to share a high number of overlapping gene members. We found that 92 of the 152 significant gene sets shared gene members with at least one other gene set. To reduce redundancy, we merged the 92 overlapping gene sets into 13 independent supersets (Table 1). Interestingly, the canonical pathways tended to merge with canonical pathways, and co-expression modules tended to merge with co-expression modules, suggesting that the two types of gene sets exhibited different biological properties. The supersets represented diverse biological pathways, including the adaptive immune system, complement cascade, cell cycle, viral infection, protein folding, RNA polymerase I/III, signalling by GPCR, signalling by Notch, activation of the GABA B receptor, B cell receptor signalling, and tRNA aminoacylation. The other 60 non-overlapping gene sets were kept intact, giving a total of 73 non-overlapping supersets. We ran a second round of MSEA to confirm that all 73 non-overlapping supersets, including 44 supersets derived from canonical pathways (Fig. 3a and ESM Table 10) and 29 from co-expression modules (Fig. 3b), retained statistical significance in our combined cohort 1 and cohort 2 datasets. Across the studied tissues, immune and cell cycle-related processes were consistently enriched, particularly in monocytes, macrophages, lymphocytes, blood, adipose tissue and pancreas.

**Table 1:** Top pathways associated with type 1 diabetes identified across multiple tissues at an FDR <5%

Supersets	Superset gene number	Tissues^a^	Pathways
Type 1 diabetes positive control	60	1, 2, 3, 4, 5, 6, 7	Positive control gene set for type 1 diabetes
S1: complement cascade	75	1, 2, 3, 4, 6, 7	Complement cascade; initial triggering of complement
S2: HIV infection	222	2, 3, 4, 6	HIV infection; host interactions of HIV factors
S3: signalling by Notch	122	1, 2, 3, 6, 7	Signalling by Notch; pre-Notch transcription and translation; pre-Notch expression and processing
S4: protein folding	53	1, 2, 4, 5, 6, 7	Protein folding; chaperone-mediated protein folding; association of TriC/CCT with target proteins during biosynthesis
S5: signalling by the B cell receptor	170	3, 4, 6, 7	Signalling by the B cell receptor; downstream signalling events of the B cell receptor
S6: RNA polymerase I, RNA polymerase III and mitochondrial transcription	95	3, 4, 6	RNA polymerase I, RNA polymerase Iii and mitochondrial transcription; RNA polymerase I transcription; RNA polymerase I promoter clearance; RNA polymerase I chain elongation
S7: RNA polymerase II transcription elongation	43	3, 4	RNA polymerase II transcription elongation; Tat-mediated elongation of the HIV-1 transcript; formation of the HIV elongation complex in the absence of HIV Tat; formation of the HIV-1 elongation complex containing HIV-1 Tat; formation of the RNA polymerase II elongation complex
S8: adaptive immune system	197	1, 3, 4, 6, 7	Adaptive immune system; class I MHC-mediated antigen processing and presentation
S9: tRNA aminoacylation	46	1, 2, 3, 4, 5, 7	tRNA aminoacylation; aminoacyl-tRNA biosynthesis; mitochondrial tRNA aminoacylation
S10: M phase	161	3, 4	Mitotic M–M/G_1_ phases; mitotic metaphase and anaphase; cell cycle; cell cycle, mitotic; M phase; mitotic anaphase
S11: signalling by GPCR	230	2, 4, 6	Signalling by GPCR; GPCR downstream signalling; GPCR ligand binding
S12: GABA receptor activation	54	1, 2, 4, 6	GABA receptor activation; activation of GABA B receptors; activation of GABA B receptors
S13: S phase	156	2, 3, 4, 6, 7	S phase, mitotic G_1_–G_1_/S phases; cell-cycle checkpoints; DNA replication, G_1_/S transition; synthesis of DNA, M/G_1_ transition; DNA replication pre-initiation; regulation of mitotic cell cycle; APC/C-mediated degradation of cell-cycle proteins; regulation of APC/C activators between G_1_/S and early anaphase

^a^Tissue codes are: 1, blood; 2, macrophage; 3, monocyte; 4, pancreas; 5, subcutaneous adipose tissue; 6, visceral omentum adipose tissue; 7, lymphocyte

### Pathways and co-expression modules based on pancreatic islet eQTLs were highly consistent between the two cohorts

**Fig. 4:**
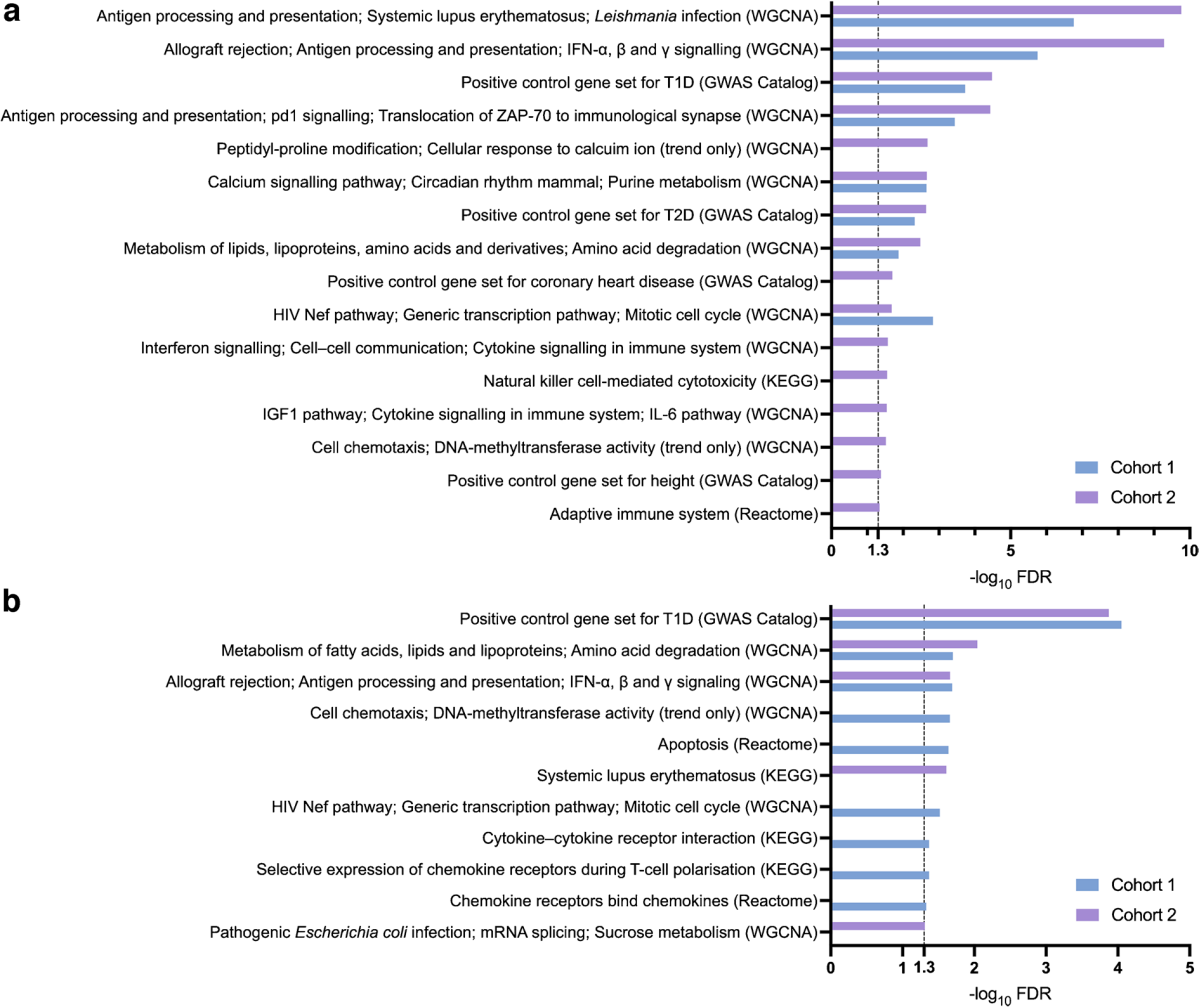
Statistically significant pancreatic islet MSEA results (FDR <0.05) from the cohort 1 and cohort 2 datasets for the canonical pathways and co-expression network modules in (**a**) pancreatic islet tissue-specific analyses, and (**b**) pancreatic islet non-HLA tissue-specific analyses for each cohort independently. ‘Trend only’ signifies that the given WGCNA module label did not pass an FDR<0.05 but was labelled with the most highly ranked pathway label match

As the pancreatic islet is the primary site of destruction in type 1 diabetes pathogenesis, we investigated this tissue separately to capture the unique perturbed pathways. From the 1827 canonical pathways and the 272 co-expression modules we evaluated, we found a total of eight pathways to be significant in cohort 1 (ESM Table 11) and 16 to be significant in cohort 2 (ESM Table 12). The eight pathways uncovered in cohort 1 included antigen presentation, calcium signalling, metabolism, transcriptional control and the type 1 diabetes positive control gene set. All of these pathways were significant in cohort 2 with stronger statistical significance. The eight pathways that were unique to cohort 2 included genes associated with coronary heart disease, immune pathways, height and insulin receptor signalling. We note that it is important to interpret pathway labels with caution. For example, the height pathway contains many immune or growth factor signalling genes that are relevant to type 1 diabetes pathogenesis. Given the smaller population size of cohort 1 and the limited availability of islet eQTL expression data (which reduce statistical power), we kept all 16 pathways uncovered in cohort 2 in our downstream analysis (including the eight pathways replicated in cohort 1) (Fig. 4a).

To gain further cell-type insight, we curated beta cell eQTLs [53] and repeated the MSEA (ESM Table 13). In both cohorts, no pathways reached significance at FDR <5% (ESM Table 14). The absence of significant signals probably reflects the limited number of beta cell eQTLs, which is attributable to the small sample size of the beta cell eQTL dataset (*n*=26 donor samples).

### The effect of removal of HLA genes on biological pathways and modules enriched for type 1 diabetes GWAS signals

As most of the known biology involved in type 1 diabetes pathogenesis is concerned with the MHC region, specifically the class I and II (HLA) genes, we hypothesised that these genes may overshadow additional important processes involved in disease pathology [46, 63–65]. We therefore performed an additional MSEA analysis that excluded the HLA genes from the 1872 canonical pathways and 272 co-expression modules.

We first examined the non-HLA MSEA results for pancreatic islets in cohort 1 and cohort 2, identifying nine significant pathways/modules in cohort 1 (ESM Table 15) and five significant pathways/modules in cohort 2 (ESM Table 16), with three that overlapped between cohorts: metabolism of lipids, antigen processing and presentation, and the positive control gene set for type 1 diabetes (Fig. 4b). Other significant pathways in cohort 1 included apoptosis, the cell cycle and immune-related pathways (e.g. the HIV pathway, cytokine receptor interaction, and chemokine receptor binding). The two unique pathways in cohort 2 were systematic lupus erythematosus and pathogenic *Escherichia coli* infection.

We then focused our non-HLA MSEA analysis on the seven informative tissues (blood, lymphocytes, macrophages, monocytes, pancreas, subcutaneous adipose tissue and visceral omentum adipose tissue). We found a total of 166 gene sets to be significant (FDR<5%) in both cohort 1 (ESM Table 15) and cohort 2 (ESM Table 16); these were also significant in meta-MSEA across both cohorts. After using our merging algorithm to reduce redundancy, we discovered a total of 64 non-overlapping supersets (ESM Table 17). Comparing these supersets to the 73 non-overlapping supersets in our results from the HLA inclusion analysis above, we observed that 39 supersets were preserved when HLA genes were removed. These include immune pathways (e.g. the adaptive immune system, cytokine signalling, and signalling by B cell receptors), insulin signalling, tRNA aminoacylation, apoptosis, protein folding and the complement cascade.

**Fig. 5:**
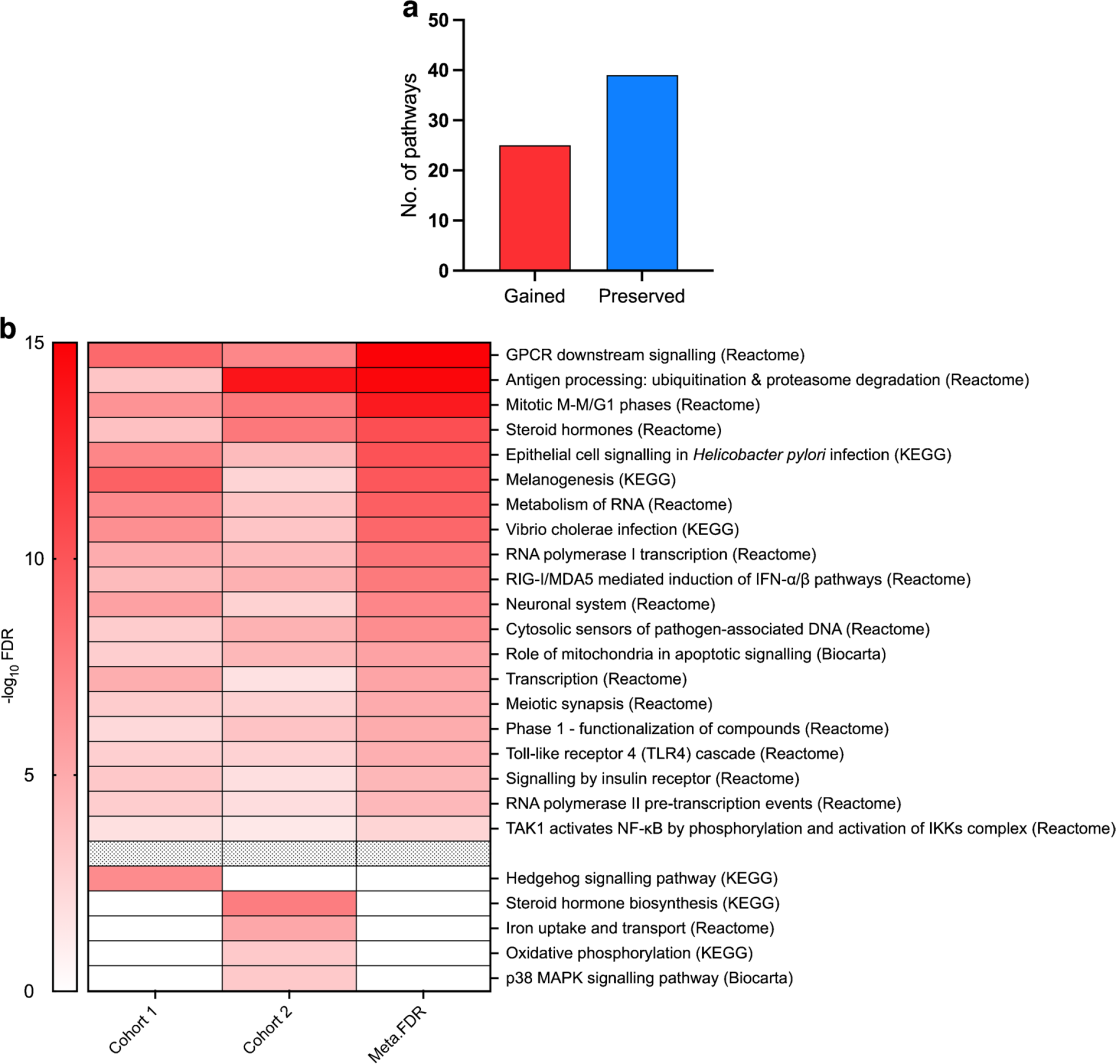
Number of preserved and gained pathways after removing the ‘HLA effect’. (**a**) Number of gained vs preserved pathways (FDR<5%). (**b**) Heatmap for gained pathways for both cohort 1 and cohort 2 above the grey cells (FDR<5%), and the pathways that were significant (FDR<5%) in either cohort 1 or cohort 2 but not both

Importantly, removal of HLA genes uncovered 25 unique supersets that were absent in our HLA inclusion results (ESM Table 17). These supersets included immune-related pathways such as antigen processing and RIG-I/MDA5 induction of IFN pathways, together with more diverse pathways including melanogenesis, steroid hormone biosynthesis, iron uptake and transport, and the role of mitochondria in apoptotic signalling (Fig. 5).

### Identification of central regulators for type 1 diabetes via a KDA

**Fig. 6:**
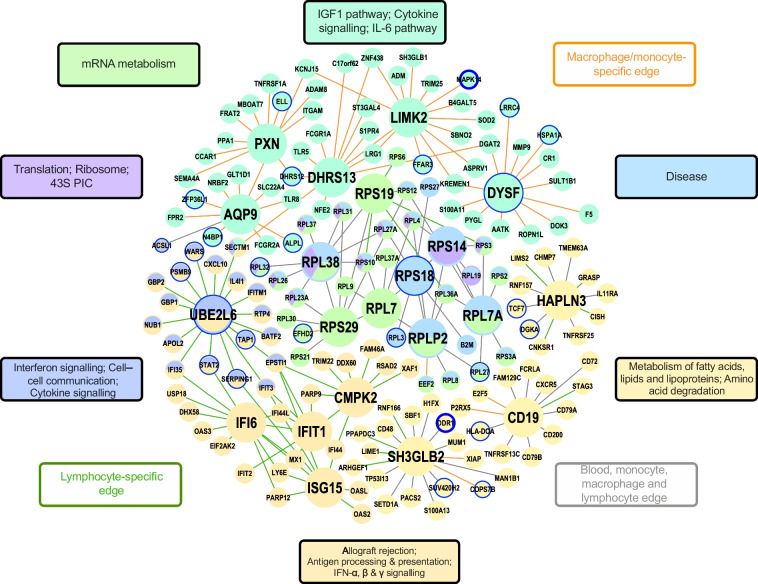
Bayesian GRNs. Combined immune-tissue GRN (blood, macrophages, monocytes, lymphocytes) inferred using a Bayesian ensemble (RIMBANet) [55] with tissue-matched expression, transcription factor/motif priors and cis-eQTL anchors. Blue circles indicate *p*<5×10^–6^ in GWAS; a larger node size indicates a KD gene; coloured nodes represent member genes; edge connections between nodes highlight the specific cell/tissue that the connections are relevant to (green: lymphocyte, orange: macrophage/monocyte, grey: blood, monocyte, macrophage and lymphocyte)

**Fig. 7:**
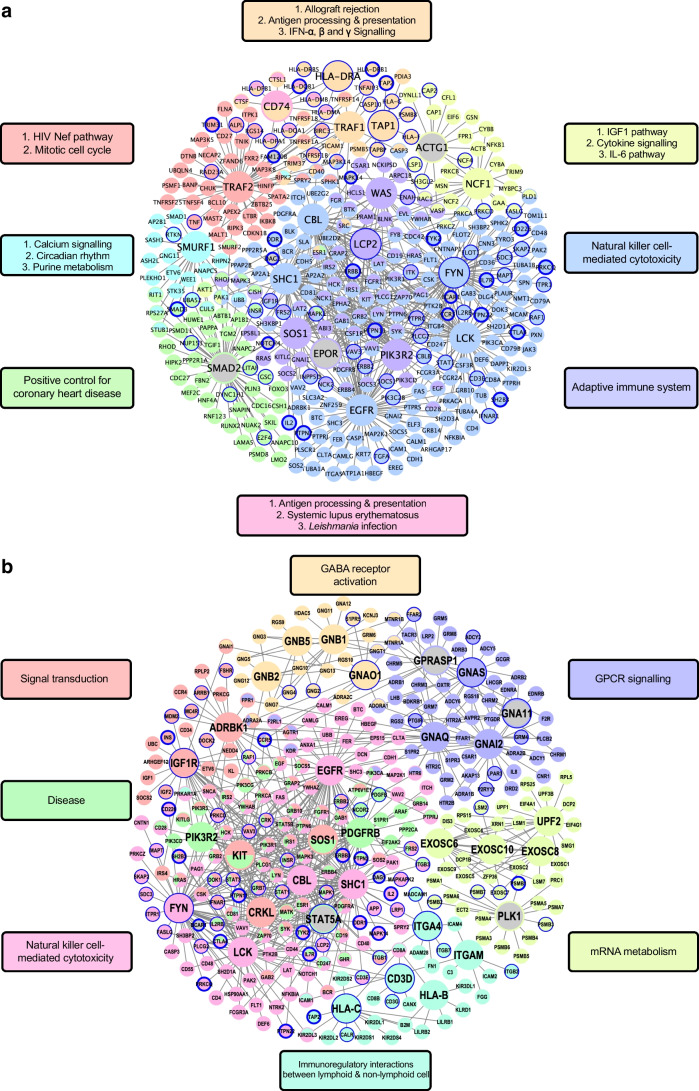
PPI networks. (**a**) Islet PPI network constructed from genes in islet-enriched pathways (MSEA, FDR<0.05) and high-confidence curated physical interactions. Edges are undirected PPIs; node size reflects connectivity degree. (**b**) Lymphocyte PPI network built as in (**a**), highlighting immune signalling hubs (e.g. LCK, FYN, CRKL, SOS1/2, STAT5) and modules for GPCR signalling, mRNA metabolism, natural killer cell cytotoxicity and signal transduction. Blue circles indicate *p*<5×10^–6^ in GWAS; a larger node size indicates a KD gene; grey nodes represent non-member genes (not derived from GWAS data); coloured nodes represent member genes

To identify central regulatory genes of the type 1 diabetes-associated gene sets uncovered by MSEA, we performed a KDA to identify KDs whose network neighbours are enriched for the type 1 diabetes-associated gene sets using PPI and tissue-specific Bayesian GRNs [54, 66] (Figs 6 and 7, and ESM Fig. 2).

#### Gene regulatory networks

We first used the islet Bayesian network due to the importance of islets in type 1 diabetes. As the islet network is sparser than the other tissue networks, probably due to the smaller sample size of available islet datasets and the highly specialised transcriptional programmes of islet cells compared with more heterogeneous tissues, we also used a combined network for islets and the hypothalamus (due to their similarity in expression patterns to those of the islet) [67]. The islet-specific network highlighted KD candidates including *TREM2*, *LY86* and *C1QC* under antigen processing and presentation, and *PARP14* and *PSMB8*, which are related to IFN signalling. These KDs were also present in the combined hypothalamic/islet network, but additionally we found KDs such as *CTSS* (involved in inflammation and antigen presentation), *CFB* (associated with the complement and coagulation cascade) and *RTP4* (under antigen processing and presentation) in the combined network (ESM Fig. 2a).

Next, focusing on the other informative tissues outside of the islet, we found a total of 348 unique KDs from our Bayesian network across the adipose tissue, blood, macrophage, monocyte, lymphocyte, and pancreas networks (FDR <5%) (ESM Table 18). To focus on the most central regulators, we chose the top five ranked KDs satisfying an FDR <5% for each type 1 diabetes-associated superset in each tissue-specific network. Among these, 17 were shared among at least two supersets and two KDs (*RPS29* and *RPS18)* were shared across the adipose tissue (including both visceral omentum and subcutaneous), blood, lymphocyte, monocyte and macrophage networks. Among the tissue networks, the adipose tissue network revealed the largest number of 47 KDs (ESM Fig. 2b), of which ten were shared with KDs uncovered in the blood, lymphocyte, monocyte and macrophage networks (Fig. 6). The uncovered KDs are related to cell cycle, metabolism and immune pathways. Notably, many of the KDs (e.g. *RPS29*, *RPLP2*, *CD19*, *IFIH1* and *PTPN6*) have been found to be involved in viral infections and autoimmune and childhood-onset diseases, all of which are associated with type 1 diabetes [68–70].

#### PPI networks

To complement the results from using GRNs and to expand our search for KDs, we performed a KDA using the tissue-specific type 1 diabetes pathways and PPI networks from the STRING database [54]. Using the islet pathways, we identified top KDs such as SMAD2, CD74, LCK, FYN, SHC1, EGFR, PIK3R2, TAP1 and HLA-DRA (Fig. 7a and ESM Table 19). For adipose tissue, blood, lymphocytes, monocytes, macrophages and pancreatic tissues (ESM Table 20), we found a total of 614 KDs. Representative KDs identified in lymphocytes included ITGAM, IGF1R, SOS1, CBL, LCK, FYN and STAT5A (Fig. 7b). Representative KDs identified in the adipose tissue included CASP9, FLNA, BRF1, CD74 and HLA-DRA, while PTPRC and MYC were identified as top KDs in blood and POLR2G and PROS1 were identified as top KDs in macrophages.

When comparing KDs from the PPI network with those from the Bayesian GRN networks, we found 26 overlapping KDs for tissue-specific networks (24 for adipose tissue, two in blood and other tissues). The top five shared KDs (FDR<5%) from both types of networks are *LCK*, *VAV1* and *PTPN6* for natural killer cell cytotoxicity and *F2* and *PLG* for complement and coagulation pathways. Other KDs include immune-related genes such as *CD19* and *CD74*, and DNA replication genes such as *MCM2* and *MCM6*, as well as those linked to autoimmunity and inflammatory responses, such as *STAT1*, *SERPING1* and *GZMB*.

#### Identification of KDs of non-HLA-specific supersets using PPI networks

As the 20 additional pathways gained from the HLA-excluded analysis were found to be significant across numerous tissues, we chose to perform a KDA using a PPI network rather than performing a tissue-specific analysis through Bayesian networks (ESM Fig. 3). We found a total of 33 unique KDs, some of which have previously been linked to type 1 diabetes, including CASP3 and CASP9 as KDs for the role of mitochondria in apoptotic signalling pathway, as well as TBP for the transcription pathway. Several KDs have also been studied in relation to type 2 diabetes and insulin resistance, including AKT1 and FGFR1 in the signalling by insulin receptor pathway, CTNNB1 in the Hedgehog signalling and melanogenesis pathway, IKBKB in the RIG-I/MDA5 induction of IFN pathways, and CHUK in the cytosolic sensors of pathogen-associated DNA pathway. Additional KDs of interest include RNF11 for antigen processing, together with RAF1 and EGFR for melanogenesis. These findings suggest that, after HLA removal, robust regulatory hubs contributing to metabolic, apoptotic and immune pathways can be identified, which are less attributable to HLA genetic contributions to type 1 diabetes.

### In silico validation of the islet KDs

**Fig. 8:**
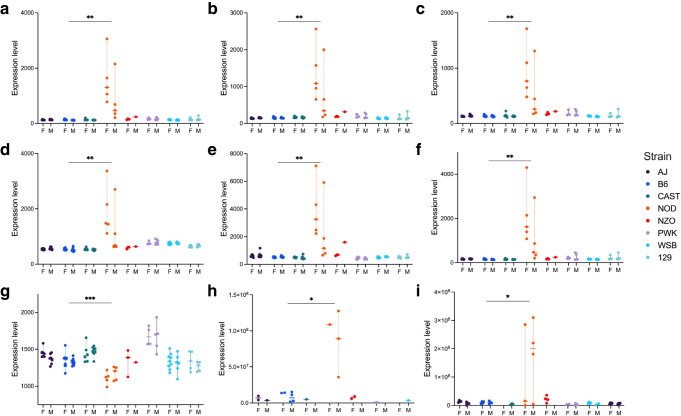
In silico validation by screening for KD gene RNA expression and proteomics patterns across the six non-diabetic mice and the one type 2 diabetic (NZO) mouse in comparison with type 1 diabetic NOD mice. The RNA expression level of the islet PPI KDs *Traf1* (**a**), *Lcp2* (**b**), *Was* (**c**), *Fyn* (**d**), *Tap1* (**e**) and *Lck* (**f**) was significantly higher in NOD mice. The RNA expression level of the islet PPI KD *Sos1* (**g**) was significantly lower in NOD mice. The protein expression level of the islet PPI KDs CD74 (**h**) and TAP1 (**i**) was significantly higher in NOD mice. *p<0.05; **p<0.01; ***p<0.001 between NOD and B6 mice. F, female mice; M, male mice

To cross-validate the KDs for specific relevance to type 1 diabetes, we examined the islet RNA sequencing and proteomics profiles from the Attie Lab Diabetes Database, in which eight founder strains of mice, such as the B6 strain and the NOD mouse (an autoimmune type 1 diabetes model), are profiled [56–59, 71]. Among the islet PPI KDs, most were upregulated in NOD mice compared with the seven non-diabetic mouse strains (Fig. 8a–f and ESM Fig. 4a–f), except for downregulation of *SOS1* (Fig. 8g). Additionally, in female NOD mice, which are more susceptible to type 1 diabetes, KD genes such as *Was*, *Traf1*, *Lcp2*, *Fyn*, *Lck*, *Cd74* and others showed higher gene expression in islet profiles. Furthermore, at the protein level, KDs CD74 and TAP1 exhibited significantly higher protein expression in NOD mice compared with the non-diabetic strains (Fig. 8h, i). Within the islet Bayesian network, a majority of the 12 KDs, including *Ctss*, *Mpeg1*, *Psmb8*, *C1qb*, *Fscn1* and *Gpb4*, were found to be significantly upregulated in NOD mice (ESM Fig. 4g–l). Therefore, the network KDs showed relevance to type 1 diabetes in independent mouse model data.

We note that, while expression of many KD genes was elevated in NOD mice, this could reflect either type 1 diabetes-specific processes or generalised inflammation during insulitis. However, another mouse strain with inflammatory phenotypes, NZO mice, did not show the same pattern of islet upregulation for the prioritised KDs, suggesting the KDs may not reflect general inflammation but are likely to be specific to autoimmunity-driven type 1 diabetes. To further assess whether the KDs and associated networks are driven by the MHC, we compared our results with a previous study [60] that compared gene expression profiles of pancreatic islets in prediabetic 2–3-week-old NOD mice and NOR mice. NOR mice serve as a diabetes-free, MHC II-matched control for NOD mice [61], allowing separation of MHC effects from other processes. In our islet PPI network, we found two KDs that overlap with their DEGs: *Cbl* and *Sos1*. *Cbl* is part of the natural killer cell-mediated cytotoxicity module, which has four KD-neighbour nodes that also overlap with the NOD vs NOR DEGs: *Plcg1*, *Ptprj*, *Sos2* and *Ube2d2*. *Sos1*, a hub gene of the adaptive immune system, together with one network neighbour gene (*Plcg1*), also overlaps with the NOD vs NOR DEGs. These findings suggest that at least some KDs and their network genes are not MHC-driven, and may represent broader regulatory mechanisms that are relevant to islet autoimmunity in type 1 diabetes.

### Drug repositioning using network KDs identified drug candidates

**Fig. 9:**
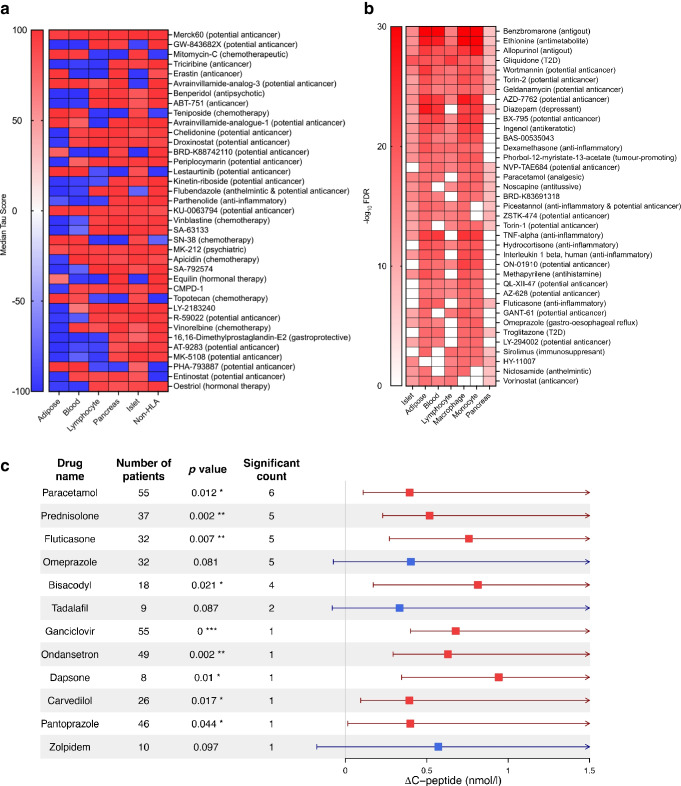
Drug repositioning results. (**a**) Heatmap showing repositioning of the top drugs using LINCS L1000 based on the KD genes for each tissue/analysis. All drugs collectively passed a median tau threshold of 90 using the absolute mean across all groups. The highest median tau score is presented in order from top to bottom. (**b**) Heatmap showing repositioning of the top drugs using PharmOmics based on the KD genes for each tissue. Only the top 100 significant drugs were included for each tissue, and common drugs with more than five appearances across all tissues are shown in the heatmaps. (**c**) Forest plot showing the individual drugs evaluated in EMR data for their association with improvement in C-peptide levels in individuals with type 1 diabetes (higher C-peptide 1 year after drug use compared with 1 year before drug use). Each drug is represented with its effect size estimate, 95% CI, and *p* value from a paired *t* test (*p<0.05; **p<0.01; ***p<0.001), together with the number of individuals included in the statistical model and the corresponding significance counts across tissues from the drug repositioning analysis. Drugs validated in (**c**) were drawn from the full predicted drug lists in ESM Tables 21 and 22

To identify drugs that could be repositioned to target gene networks associated with type 1 diabetes, we used KD genes from individual tissues as an input into the drug repositioning tools LINCS L1000 and PharmOmics [44, 45]. For results from LINCS L1000, we filtered the drugs that pass a median tau threshold of ±90 as we cannot infer directionality from our input genes (Fig. 9a and ESM Table 21). From PharmOmics, we selected the significant drugs that ranked within the top 100 in each tissue and appeared at least five times in any of the screened tissues (Fig. 9b and ESM Table 22). These complementary approaches identified several mechanistically coherent drug categories that may be broadly grouped into immune and inflammatory modulators and metabolic and stress modulators.

Within the immune and inflammatory domain, we identified apicidin, etinostat, Merck60, droxinostat and vorinostat, which are all selective HDAC inhibitors mainly targeting HDAC1, HDAC3, HDAC6 and HDAC9, as top hits in our drug search across various tissues in both PPI and Bayesian type 1 diabetes networks. The GSK-3β inhibitors kenpaullone and indirubin were also significant hits in our lymphocyte and pancreas networks, which agrees with previous literature showing that inhibition of GSK-3β protects against pancreatic beta cell death [72]. Kenpaullone is not only involved in GSK-3β inhibition but is also a CDK inhibitor and an Src inhibitor, which may provide further protection to beta cells. Additionally, the mTOR inhibitor sirolimus and the PI3K inhibitor LY-294002, which were found to be significant drugs using adipose tissue, macrophage and monocyte network KDs, also indirectly regulate GSK-3β via the PI3K–Akt–mTOR signalling axis [73].

IKK inhibitors (TPCA-1, BMS-345541, BX-795, parthenolide and IKK2 inhibitor V) were the third major drug class identified in our analysis of islet, lymphocyte, adipose tissue and non-HLA blood network KDs. IKK inhibitors are potential drug options to consider in various autoimmune diseases, such as rheumatoid arthritis, lupus erythematosus, multiple sclerosis and irritable bowel disease, through their modulation of NF-κB signalling, which is also a known key pathway in type 1 diabetes [74]. Finally, glucocorticoids (prednisolone, hydrocortisone and fluticasone) were predicted by both LINCS L1000 and PharmOmics. Glucocorticoids are well-established immunosuppressants that reduce inflammation by dampening signal transduction downstream of cytokine receptors, inhibiting cytokine production and broadly suppressing immune responses [75, 76].

In addition to these immune-related categories, our analyses also identified metabolic and stress modulators. Pain and proton modulators such as paracetamol (acetaminophen), omeprazole and pantoprazole were predicted across multiple tissues. These agents are known to influence glutathione metabolism, oxidative stress buffering and mitochondrial function, all of which are processes critical to beta cell survival in type 1 diabetes. Cardiometabolic modulators, including carvedilol and troglitazone, were also predicted by the analyses. Carvedilol, a non-selective β-blocker with antioxidant properties, improves insulin sensitivity and reduces oxidative stress, while troglitazone, a PPARγ agonist, directly enhances insulin sensitivity and glucose use. Another important metabolic category included uric acid and purine metabolism modulators, represented by allopurinol and benzbromarone, which were the top two hits found via PharmOmics. These agents lower uric acid levels through either xanthine oxidase inhibition (allopurinol) or increased renal excretion (benzbromarone), which is noteworthy given the strong links between hyperuricaemia, oxidative stress, inflammation and beta cell dysfunction [77, 78]. Additionally, the anti-emetic ondansetron (a 5-HT3 receptor antagonist) and the laxative bisacodyl were also predicted.

Drugs of interest that only targeted the islet network KDs included tenofovir, PKCβ inhibitors and YM-976. All of these have been indicated for treatment of various immune and autoimmune diseases. The reverse transcriptase inhibitor tenofovir, administered as a component of the HIV medication Truvada, has been shown to improve hereditary autoimmune inflammation thought to be caused by build-up of retroelement cDNA in mice [79]. Levonorgestrel, a progesterone receptor agonist, was found to pass our drug match criteria in the LINCS L1000 for our lymphocyte KDs. Increasing progesterone levels has been found to be protective against rheumatoid arthritis and multiple sclerosis; reducing stress hormones has been shown to reduce inflammation [80], which may help delay the progression of beta cell death.

Collectively, our network-based drug repositioning captures both immune/inflammatory regulators (HDAC, GSK-3β, IKK inhibitors and glucocorticoids) and metabolic/stress modulators acting on redox, cardiometabolic and uric acid pathways. These results underscore the complementary roles of immune control and metabolic regulation in the pathophysiology of type 1 diabetes, and provide multiple mechanistically supported avenues for therapeutic intervention.

### In silico validation of predicted drugs

To validate our drug predictions, we assessed whether any predicted drugs significantly increased C-peptide levels in individuals with type 1 diabetes. C-peptide, which is a by-product of proinsulin cleavage during insulin synthesis, is released in equimolar amounts alongside endogenous insulin [70]. Thus, C-peptide is not affected by exogenous insulin therapy, making it a reliable biomarker for endogenous insulin secretion and islet function [81].

Using EMRs from the OneFlorida+ Clinical Data Network, we extracted medication histories and C-peptide levels (nmol/l) for individuals with type 1 diabetes. For each drug, we compared the mean C-peptide levels during the year before vs the year after drug exposure. Notably, individuals with type 1 diabetes who used predicted drugs and drug classes such as paracetamol, pantoprazole and glucocorticoids (prednisolone and fluticasone) showed significantly increased C-peptide levels (Fig. 9c). Other predicted drugs that showed significance in EMR validation include bisacodyl, ondansetron, tadalafil, ganciclovir, dapsone and zolpidem. When narrowing the window to 6 months before and after exposure, only prednisolone, carvedilol, ondansetron and bisacodyl remained significant among the drugs shown in Fig. 9c. By contrast, when extending the window to 2 years, the drugs shown in Fig. 9c remained or became significant except for bisacodyl and tadalafil. These findings suggest that these drugs may have potential therapeutic benefits in improving pancreatic islet function in individuals with type 1 diabetes.

## Discussion

Previous genetic studies have uncovered at least 60 loci linked with type 1 diabetes development, yet our understanding of the intricate mechanisms underlying these associations and GWAS-based intervention approaches remains limited. To this end, we used a multi-omics integrative approach to advance our understanding of type 1 diabetes aetiology and prioritise potential therapeutic targets among the large number of disease-associated signals. The dysregulated pathways and key regulators of type 1 diabetes aetiology were elucidated by combined analysis of the full summary statistics of two independent type 1 diabetes GWAS datasets, functional genomics data, knowledge-driven pathways, and data-driven gene co-expression and regulatory networks. This approach enabled identification of both well-known immune regulators and novel candidates, several of which showed increased expression specifically in islets of diabetic NOD mice, supporting their functional relevance. We further used the networks to guide drug repositioning, identifying drug candidates that target the type 1 diabetes networks, some of which were validated in an EMR cohort.

We identified a significant number of networks involved in immune and apoptosis-related processes that have previously been implicated in type 1 diabetes [82]. We also found several pathways that have previously been considered to be less associated with the genetic contribution of type 1 diabetes, such as viral infection, Notch signalling, amino acid degradation, and the IGF-1 and insulin signalling pathways [75, 83]. Our tissue-specific analyses revealed a consistent enrichment of antigen processing/presentation, natural killer cell cytotoxicity and IFN-α/β/γ signalling pathways across multiple tissues, supporting the immune origin of type 1 diabetes highlighted in the literature, and emphasising the presence of systemic dysregulation of immune function beyond insulitis [84].

In addition to the classic immune components, we identified several pathways that imply a pathogenic change in the molecular machinery coordinating protein production and processing, including the spliceosome in monocytes, mRNA metabolism in blood, macrophages and monocytes, tRNA aminoacylation in macrophages, monocytes and subcutaneous adipose tissue, and proteasome in blood, macrophages and monocytes. Kracht et al reported that production of a non-conventional insulin-derived product due to alternative translation of the insulin transcript results in a polypeptide that is detected by T cells and elicits an autoimmune response in individuals with type 1 diabetes [85]. It has also been predicted that the formation of hybrid insulin peptides within beta cells activates CD4 T cells in NOD mice [86], thus further supporting the association between type 1 diabetes and the failure of proteins to be processed correctly. It is plausible that variations within genes governing mRNA processing as well as protein formation and processing components induce antigenic protein products within the pancreatic tissue itself or within immune cells, resulting in activation of an autoimmune response, beta cell death and development of type 1 diabetes.

Importantly, within our tissue-specific networks, we were able to capture and highlight numerous previously implicated contributors to type 1 diabetes, either as hub genes (e.g. *IFIH1*, *HLA-C*, *SLC15A3* and *RAD51*) [87] or peripheral genes (*CTLA4*, *PTPN22*, *INS*, *INSR* and *HLA-DQA*). Of note, within the expected type 1 diabetes immune pathways such as cytokine signalling, we uncovered KD genes that are not well recognised for their specific role in type 1 diabetes but were consistent across tissues and connected to well-known type 1 diabetes GWAS hits. For example, *FYN*, a KD in both lymphocyte (Fig. 6) and islet PPI networks (Fig. 7a), was connected with type 1 diabetes GWAS loci (*CTLA4*, *INSR*, *CD226* and *PTPN11*). This gene is essential in T cell signalling and interacts with *ZAP-70* [88] and *VAV1* [89, 90], which are key components of the T cell-mediated immune response and are connected to *FYN* in our lymphocyte and islet PPI networks. Similarly, the KD LCK in the islet network has also been previously shown to inhibit autoimmune responses through its interaction with DUSP22 as a negative regulator of T cell activation [91]. Both *Fyn* and *Lck* showed increased expression specifically in NOD mouse islets compared with mouse models of type 2 diabetes and non-diabetes (Fig. 7).

Interestingly, in less well-studied tissues for type 1 diabetes such as adipose tissue, we captured previously documented type 1 diabetes genes such as *SLC15A3* and *IFIH1.* Our network analysis suggests a role for *SLC15A3* in signal transduction in adipose tissue, including the regulation of inflammatory signals [92]. *IFIH1*, a player in the innate immune response, is triggered by viral infections and is a regulator of the diabetogenic T cell response [93].

Viral infection and its association with type 1 diabetes have been suggested as the potential causal environmental trigger, particularly antenatal maternal infection and the subsequent incidence of type 1 diabetes [94]. In support of this, we found several pathways associated with HIV and influenza virus infection across multiple tissues tested. Given that these pathways are genetically perturbed as informed by type 1 diabetes GWAS datasets, our finding implies that genetic variants in genes involved in viral infection may confer vulnerability to infections and/or promote an over-reactive viral response that induces autoimmunity, which may explain how viral infection triggers type 1 diabetes pathogenesis. Moreover, we found numerous KDs that are known to have a link with viruses but for which there is limited evidence for direct genetic association with type 1 diabetes, such as *Ifit1*, which has been shown to be induced in NOD mice after rotavirus infection [95], *Oas2*, which encodes an innate immune-activated antiviral enzyme [96], and *Isg15*, which encodes an antiviral effector linked to anti-apoptosis in MIN6 cells [97]. These genes were found to be within our blood and lymphocyte GRNs under the IFN signalling pathway, and were KDs in our type 1 diabetes adipose tissue network.

To capture additional regulatory pathways and KDs that may have been overshadowed by the ‘HLA effect’, we ran pathway and network analyses after removing HLA-related genes. These analyses captured pathways that may contribute to type 1 diabetes through gene–environment interactions, such as viral infection pathways (including RIG-I/MDA5 induction, *IFIH1-*related genes and cytosolic sensors of pathogen-associated DNA), bacterial infection pathways such as epithelial cell signalling in *Helicobacter pylori* infection, melanogenesis (which may link a lack of vitamin D with type 1 diabetes development), and insulin receptor signalling, which may interact with any or a mixture of the above factors.

Our in silico validation of islet KDs highlights their significant role in the NOD mouse. The upregulation of KDs such as *Ctss*, which when translated degrades antigenic proteins [98], *Cfb*, which contributes to complement activation [99], and *Psmb8*, induced by IFN-γ [100], underscores their potential involvement in type 1 diabetes pathogenesis. The observed sex-specific expression, especially in female NOD mice, further supports the potential role of these KDs in the heightened susceptibility to type 1 diabetes development in female individuals. The elevated protein expression of the immune-related KD genes *Cd74* and *Tap1* in NOD mice compared with other strains could indicate a more pronounced immune response, which aligns with the autoimmune nature of type 1 diabetes. These orthogonal validation results offer support for these KDs in type 1 diabetes pathogenesis, and may inform future therapeutic strategies.

Collectively taking into account all the type 1 diabetes-associated pathways, KDs and tissues, we next aimed to find compounds that could be used to target these dysfunctional gene networks, using the LINCS L1000 and PharmOmics drug repositioning tools [44, 45]. We observed two complementary therapeutic themes: (1) immune/inflammatory modulators targeting core signalling nodes implicated in beta cell stress and autoimmunity (e.g. HDAC-, GSK-3β- and IKK/NF-κB-linked mechanisms), and (2) metabolic/stress modulators predicted to support beta cell resilience through redox, mitochondrial and broader cardiometabolic pathways. This dual pattern is consistent with the established view that type 1 diabetes progression reflects both immune-mediated injury and beta cell-intrinsic vulnerability, and suggests that multi-pronged strategies that combine immune control with metabolic support may be particularly relevant for preserving endocrine function. Importantly, several predicted compounds that are commonly used clinically showed concordant signals in our OneFlorida+ EMR validation based on pre-treatment/post-treatment changes in C-peptide. In particular, we obtained evidence supporting representative hits from both thematic groups (anti-inflammatory/immunomodulatory agents [glucocorticoids such as prednisolone and fluticasone] and metabolic/stress modulators [e.g. paracetamol and proton pump inhibitors such as pantoprazole/omeprazole]), with additional supportive signals in sensitivity analyses for carvedilol and select gastrointestinal supportive agents (ondansetron and bisacodyl). Together, these overlaps provide a coherent bridge between in silico network targeting and real-world biomarker shifts consistent with preserved endogenous insulin secretion. Additional drug predictions, particularly for strong immune modulators, may not have appeared in the EMRs due to their limited use in the patient population.

While our integrative approach revealed numerous candidate genes, pathways and drug targets, limitations should be noted. The findings are based primarily on in silico analyses and EMR data, and experimental validation in animal models and human tissues will be essential to establish causality and therapeutic efficacy. Bayesian GRNs infer directionally informed statistical dependencies rather than definitive causality, which requires experimental or clinical perturbation for validation. In addition, we also performed beta cell eQTL-based pathway analysis but found no significant pathway enrichments in either cohort. This probably reflects limited power, sparser SNP-to-gene links after LD pruning, and the use of basal beta cell profiles that may miss context-dependent regulation. Accordingly, larger, type 1 diabetes-specific and single-cell-based beta cell eQTL resources are needed to assess beta cell-intrinsic pathways with adequate power. Similarly, incorporating well-powered disease-specific islet eQTL datasets (when available) will be important for improving such analyses. Additionally, while stage-wise FDR control in sequential designs is expected to be conservative, extending pipeline-wide FDR control to our multi-stage framework remains an important direction for future analyses to further limit false positives. Lastly, we note that cohort-level attributes (e.g. BMI, age, sex and environmental exposures) could contribute to differences between the GWAS cohorts, but detailed metadata were not available to evaluate this.

Overall, our multi-tissue multi-omics integrative analysis recapitulates previously known pathways and genes associated with type 1 diabetes pathogenesis, confirming the validity of the approach. We additionally uncovered novel pathways and key genes in multiple tissues that potentially contribute to type 1 diabetes development through genetic perturbations, including those that can interact with environmental factors. These findings offer a comprehensive understanding of type 1 diabetes pathogenesis based on genetic evidence and through an omnigenic network lens. The KDs prioritised through our comprehensive integrative analyses may serve as putative therapeutic targets for type 1 diabetes, and, together with our drug predictions, if experimentally validated in future studies, may help delay or prevent type 1 diabetes progression.

## Supplementary Information

Below is the link to the electronic supplementary material.

ESM (PDF 854 KB)

ESM Tables (XLSX 23664 KB)

## Data Availability

All data supporting the findings of this study are available within the paper and the ESM.

## Abbreviations

DEG: Differentially expressed gene
EMR: Electronic medical record
eQTL: Expression quantitative trait locus
FDR: False discovery rate
GRN: Gene regulatory network
GWAS: Genome-wide association studies
KD: Key driver
KDA: Key driver analysis
LD: Linkage disequilibrium
MSEA: Marker set enrichment analysis
NOR: Non-obese resistant
PPI: Protein–protein interaction

## References

[1] Maahs DM, West NA, Lawrence JM, Mayer-Davis EJ (2010) Epidemiology of type 1 diabetes. Endocrinol Metab Clin 39(3):481–497. 10.1016/j.ecl.2010.05.011 10.1016/j.ecl.2010.05.011 PMC2925303 20723815

[2] Mobasseri M, Shirmohammadi M, Amiri T, Vahed N, Fard HH, Ghojazadeh M (2020) Prevalence and incidence of type 1 diabetes in the world: a systematic review and meta-analysis. Health Promot Perspect 10(2):98. 10.34172/hpp.2020.18 32296622 10.34172/hpp.2020.18 PMC7146037

[3] Ogrotis I, Koufakis T, Kotsa K (2023) Changes in the global epidemiology of type 1 diabetes in an evolving landscape of environmental factors: causes, challenges, and opportunities. Medicina (Mex) 59(4):668. 10.3390/medicina59040668 10.3390/medicina59040668 PMC10141720 37109626

[4] Steck AK, Barriga KJ, Emery LM, Fiallo-Scharer RV, Gottlieb PA, Rewers MJ (2005) Secondary attack rate of type 1 diabetes in Colorado families. Diabetes Care 28(2):296–300. 10.2337/diacare.28.2.296 15677782 10.2337/diacare.28.2.296

[5] Khamis A, Canouil M, Siddiq A et al (2019) Laser capture microdissection of human pancreatic islets reveals novel eQTLs associated with type 2 diabetes. Mol Metab 24:98–107. 10.1016/j.molmet.2019.03.004 30956117 10.1016/j.molmet.2019.03.004 PMC6531807

[6] Oilinki T, Otonkoski T, Ilonen J, Knip M, Miettinen P (2012) Prevalence and characteristics of diabetes among Somali children and adolescents living in Helsinki, Finland. Pediatr Diabetes 13(2):176–180. 10.1111/j.1399-5448.2011.00783.x 21595807 10.1111/j.1399-5448.2011.00783.x

[7] Söderström U, Åman J, Hjern A (2012) Being born in Sweden increases the risk for type 1 diabetes – a study of migration of children to Sweden as a natural experiment. Acta Paediatr 101(1):73–77. 10.1111/j.1651-2227.2011.02410.x 21767306 10.1111/j.1651-2227.2011.02410.x

[8] Kostic AD, Gevers D, Siljander H et al (2015) The dynamics of the human infant gut microbiome in development and in progression toward type 1 diabetes. Cell Host Microbe 17(2):260–273. 10.1016/j.chom.2015.01.001 25662751 10.1016/j.chom.2015.01.001 PMC4689191

[9] Norris JM, Barriga K, Klingensmith G et al (2003) Timing of initial cereal exposure in infancy and risk of islet autoimmunity. JAMA 290(13):1713–1720. 10.1001/jama.290.13.1713 14519705 10.1001/jama.290.13.1713

[10] Weets I, Kaufman L, Van Der Auwera B et al (2004) Seasonality in clinical onset of type 1 diabetes in Belgian patients above the age of 10 is restricted to HLA-DQ2/DQ8-negative males, which explains the male to female excess in incidence. Diabetologia 47:614–621. 10.1007/s00125-004-1369-8 15298337 10.1007/s00125-004-1369-8

[11] Gamble D, Kinsley M, FitzGerald M, Bolton R, Taylor K (1969) Viral antibodies in diabetes mellitus. Br Med J 3(5671):627–630. 10.1136/bmj.3.5671.627 5811681 10.1136/bmj.3.5671.627 PMC1984442

[12] Coppieters K, Wiberg A, Tracy S, Von Herrath M (2012) Immunology in the clinic review series: focus on type 1 diabetes and viruses: the role of viruses in type 1 diabetes: a difficult dilemma. Clin Exp Immunol 168(1):5–11. 10.1111/j.1365-2249.2011.04554.x 22385231 10.1111/j.1365-2249.2011.04554.x PMC3390487

[13] Yeung WCG, Rawlinson WD, Craig ME (2011) Enterovirus infection and type 1 diabetes mellitus: systematic review and meta-analysis of observational molecular studies. BMJ 342:d35. 10.1136/bmj.d35 21292721 10.1136/bmj.d35 PMC3033438

[14] Blanter M, Sork H, Tuomela S, Flodström-Tullberg M (2019) Genetic and environmental interaction in type 1 diabetes: a relationship between genetic risk alleles and molecular traits of enterovirus infection? Curr Diab Rep 19:82. 10.1007/s11892-019-1192-8 31401790 10.1007/s11892-019-1192-8 PMC6689284

[15] Esposito S, Toni G, Tascini G, Santi E, Berioli MG, Principi N (2019) Environmental factors associated with type 1 diabetes. Front Endocrinol 10:592. 10.3389/fendo.2019.00592 10.3389/fendo.2019.00592 PMC6722188 31555211

[16] Zorena K, Michalska M, Kurpas M, Jaskulak M, Murawska A, Rostami S (2022) Environmental factors and the risk of developing type 1 diabetes – old disease and new data. Biology 11(4):608. 10.3390/biology11040608 35453807 10.3390/biology11040608 PMC9027552

[17] Lambert AP, Gillespie KM, Thomson G et al (2004) Absolute risk of childhood-onset type 1 diabetes defined by human leukocyte antigen class II genotype: a population-based study in the United Kingdom. J Clin Endocrinol Metab 89(8):4037–4043. 10.1210/jc.2003-032084 15292346 10.1210/jc.2003-032084

[18] Noble JA, Valdes AM, Cook M, Klitz W, Thomson G, Erlich HA (1996) The role of HLA class II genes in insulin-dependent diabetes mellitus: molecular analysis of 180 Caucasian, multiplex families. Am J Hum Genet 59(5):1134 8900244 PMC1914851

[19] Noble JA, Erlich HA (2012) Genetics of type 1 diabetes. Cold Spring Harb Perspect Med 2(1):a007732. 10.1101/cshperspect.a007732 22315720 10.1101/cshperspect.a007732 PMC3253030

[20] Boyle EA, Li YI, Pritchard JK (2017) An expanded view of complex traits: from polygenic to omnigenic. Cell 169(7):1177–1186. 10.1016/j.cell.2017.05.038 28622505 10.1016/j.cell.2017.05.038 PMC5536862

[21] Krishnan KC, Kurt Z, Barrere-Cain R et al (2018) Integration of multi-omics data from mouse diversity panel highlights mitochondrial dysfunction in non-alcoholic fatty liver disease. Cell Syst 6(1):103–115. 10.1016/j.cels.2017.12.006 29361464 10.1016/j.cels.2017.12.006 PMC5799036

[22] Kurt Z, Cheng J, Barrere-Cain R et al (2023) Shared and distinct pathways and networks genetically linked to coronary artery disease between human and mouse. eLife 12:RP88266. 10.7554/eLife.88266 38060277 10.7554/eLife.88266 PMC10703441

[23] Shu L, Zhao Y, Kurt Z et al (2016) Mergeomics: multidimensional data integration to identify pathogenic perturbations to biological systems. BMC Genomics 17:872. 10.1186/s12864-016-3198-9 27814671 10.1186/s12864-016-3198-9 PMC5097440

[24] Shu L, Chan KHK, Zhang G et al (2017) Shared genetic regulatory networks for cardiovascular disease and type 2 diabetes in multiple populations of diverse ethnicities in the United States. PLoS Genet 13(9):e1007040. 10.1371/journal.pgen.1007040 28957322 10.1371/journal.pgen.1007040 PMC5634657

[25] Blencowe M, Ahn IS, Saleem Z et al (2021) Gene networks and pathways for plasma lipid traits via multitissue multiomics systems analysis. J Lipid Res 62:100019. 10.1194/jlr.RA120000713 33561811 10.1194/jlr.RA120000713 PMC7873371

[26] Zhao Y, Blencowe M, Shi X et al (2019) Integrative genomics analysis unravels tissue-specific pathways, networks, and key regulators of blood pressure regulation. Front Cardiovasc Med 6:21. 10.3389/fcvm.2019.00021 30931314 10.3389/fcvm.2019.00021 PMC6423920

[27] Chan KHK, Huang YT, Meng Q et al (2014) Shared molecular pathways and gene networks for cardiovascular disease and type 2 diabetes mellitus in women across diverse ethnicities. Circ Cardiovasc Genet 7(6):911–919. 10.1161/CIRCGENETICS.114.000676 25371518 10.1161/CIRCGENETICS.114.000676

[28] Croft D, Mundo AF, Haw R et al (2014) The reactome pathway knowledgebase. Nucleic Acids Res 42(D1):D472–D477. 10.1093/nar/gkt1102 24243840 10.1093/nar/gkt1102 PMC3965010

[29] Ding J, Blencowe M, Nghiem T et al (2021) Mergeomics 2.0: a web server for multi-omics data integration to elucidate disease networks and predict therapeutics. Nucleic Acids Res 49(W1):W375–W387. 10.1093/nar/gkab405 34048577 10.1093/nar/gkab405 PMC8262738

[30] Alman AC, Smith SR, Eckel RH et al (2017) The ratio of pericardial to subcutaneous adipose tissues is associated with insulin resistance. Obesity 25(7):1284–1291. 10.1002/oby.21875 28558132 10.1002/oby.21875 PMC5488713

[31] Arif S, Leete P, Nguyen V et al (2014) Blood and islet phenotypes indicate immunological heterogeneity in type 1 diabetes. Diabetes 63(11):3835–3845. 10.2337/db14-0365 24939426 10.2337/db14-0365 PMC4207393

[32] Burg AR, Tse HM (2018) Redox-sensitive innate immune pathways during macrophage activation in type 1 diabetes. Antioxid Redox Signal 29(14):1373–1398. 10.1089/ars.2017.7243 29037052 10.1089/ars.2017.7243 PMC6166692

[33] Campbell-Thompson M, Rodriguez-Calvo T, Battaglia M (2015) Abnormalities of the exocrine pancreas in type 1 diabetes. Curr Diab Rep 15:79. 10.1007/s11892-015-0653-y 26318606 10.1007/s11892-015-0653-y PMC5072278

[34] Devaraj S, Glaser N, Griffen S, Wang-Polagruto J, Miguelino E, Jialal I (2006) Increased monocytic activity and biomarkers of inflammation in patients with type 1 diabetes. Diabetes 55(3):774–779. 10.2337/diabetes.55.03.06.db05-1417 16505242 10.2337/diabetes.55.03.06.db05-1417

[35] Hinman RM, Cambier JC (2014) Role of B lymphocytes in the pathogenesis of type 1 diabetes. Curr Diab Rep 14:543. 10.1007/s11892-014-0543-8 25189436 10.1007/s11892-014-0543-8

[36] Kotani R, Nagata M, Imagawa A et al (2004) T lymphocyte response against pancreatic beta cell antigens in fulminant type 1 diabetes. Diabetologia 47:1285–1291. 10.1007/s00125-004-1441-4 15243701 10.1007/s00125-004-1441-4

[37] Miao F, Smith DD, Zhang L, Min A, Feng W, Natarajan R (2008) Lymphocytes from patients with type 1 diabetes display a distinct profile of chromatin histone H3 lysine 9 dimethylation: an epigenetic study in diabetes. Diabetes 57(12):3189–3198. 10.2337/db08-0645 18776137 10.2337/db08-0645 PMC2584123

[38] Rodrigues KB, Dufort MJ, Llibre A et al (2020) Innate immune stimulation of whole blood reveals IFN 1 hyper-responsiveness in type 1 diabetes. Diabetologia 63:1576–1587. 10.1007/s00125-020-05179-4 32500289 10.1007/s00125-020-05179-4 PMC10091865

[39] Thiem K, van Dierendonck XA, Janssen AW et al (2020) A high glycemic burden relates to functional and metabolic alterations of human monocytes in patients with type 1 diabetes. Diabetes 69(12):2735–2746. 10.2337/db20-0568 32978233 10.2337/db20-0568

[40] Cooper NJ, Wallace C, Burren O, Cutler A, Walker N, Todd JA (2017) Type 1 diabetes genome-wide association analysis with imputation identifies five new risk regions. BioRxiv 120022. 10.1101/120022

[41] GTEx Consortium (2020) The GTEx Consortium atlas of genetic regulatory effects across human tissues. Science 369(6509):1318–1330. 10.1126/science.aaz1776 32913098 10.1126/science.aaz1776 PMC7737656

[42] Rotival M, Zeller T, Wild PS et al (2011) Integrating genome-wide genetic variations and monocyte expression data reveals trans-regulated gene modules in humans. PLoS Genet 7(12):e1002367. 10.1371/journal.pgen.1002367 22144904 10.1371/journal.pgen.1002367 PMC3228821

[43] Schmiedel BJ, Singh D, Madrigal A et al (2018) Impact of genetic polymorphisms on human immune cell gene expression. Cell 175(6):1701–1715. 10.1016/j.cell.2018.10.022 30449622 10.1016/j.cell.2018.10.022 PMC6289654

[44] Chen YW, Diamante G, Ding J et al (2022) PharmOmics: a species-and tissue-specific drug signature database and gene-network-based drug repositioning tool. iScience 25(4):104052. 10.1016/j.isci.2022.104052 35345455 10.1016/j.isci.2022.104052 PMC8957031

[45] Duan Q, Reid SP, Clark NR et al (2016) L1000CDS2: LINCS L1000 characteristic direction signatures search engine. NPJ Syst Biol Appl 2(1):16015. 10.1038/npjsba.2016.15 28413689 10.1038/npjsba.2016.15 PMC5389891

[46] Barrett JC, Clayton DG, Concannon P et al (2009) Genome-wide association study and meta-analysis find that over 40 loci affect risk of type 1 diabetes. Nat Genet 41(6):703–707. 10.1038/ng.381 19430480 10.1038/ng.381 PMC2889014

[47] GTEx Consortium (2015) The Genotype-Tissue Expression (GTEx) pilot analysis: multitissue gene regulation in humans. Science 348(6235):648–660. 10.1126/science.1262110 25954001 10.1126/science.1262110 PMC4547484

[48] Viñuela A, Varshney A, van de Bunt M et al (2020) Genetic variant effects on gene expression in human pancreatic islets and their implications for type 2 diabetes. Nat Commun 11(1):4912. 10.1038/s41467-020-18581-8 32999275 10.1038/s41467-020-18581-8 PMC7528108

[49] Kaestner KH, Powers AC, Naji A, HPAP Consortium, Atkinson MA (2019) NIH initiative to improve understanding of the pancreas, islet, and autoimmunity in type 1 diabetes: the Human Pancreas Analysis Program (HPAP). Diabetes 68(7):1394–1402. 10.2337/db19-0058 31127054 10.2337/db19-0058 PMC6609987

[50] Langfelder P, Horvath S (2008) WGCNA: an R package for weighted correlation network analysis. BMC Bioinformatics 9:559. 10.1186/1471-2105-9-559 19114008 10.1186/1471-2105-9-559 PMC2631488

[51] Nishimura D (2001) BioCarta. Biotech Softw Internet Rep 2(3):117–120. 10.1089/152791601750294344

[52] Kanehisa M, Goto S (2000) KEGG: Kyoto Encyclopedia of Genes and Genomes. Nucleic Acids Res 28(1):27–30. 10.1093/nar/28.1.27 10592173 10.1093/nar/28.1.27 PMC102409

[53] MacArthur J, Bowler E, Cerezo M et al (2017) The new NHGRI-EBI Catalog of published genome-wide association studies (GWAS Catalog). Nucleic Acids Res 45(D1):D896–D901. 10.1093/nar/gkw1133 27899670 10.1093/nar/gkw1133 PMC5210590

[54] Szklarczyk D, Gable AL, Nastou KC et al (2021) The STRING database in 2021: customizable protein–protein networks, and functional characterization of user-uploaded gene/measurement sets. Nucleic Acids Res 49(D1):D605–D612. 10.1093/nar/gkaa1074 33237311 10.1093/nar/gkaa1074 PMC7779004

[55] Zhu J, Zhang B, Smith EN et al (2008) Integrating large-scale functional genomic data to dissect the complexity of yeast regulatory networks. Nat Genet 40(7):854–861. 10.1038/ng.167 18552845 10.1038/ng.167 PMC2573859

[56] Mitok KA, Freiberger EC, Schueler KL et al (2018) Islet proteomics reveals genetic variation in dopamine production resulting in altered insulin secretion. J Biol Chem 293(16):5860–5877. 10.1074/jbc.RA117.001102 29496998 10.1074/jbc.RA117.001102 PMC5912463

[57] Keller MP, Gatti DM, Schueler KL et al (2018) Genetic drivers of pancreatic islet function. Genetics 209(1):335–356. 10.1534/genetics.118.300864 29567659 10.1534/genetics.118.300864 PMC5937189

[58] Dong C, Simonett SP, Shin S et al (2021) INFIMA leverages multi-omics model organism data to identify effector genes of human GWAS variants. Genome Biol 22(1):241. 10.1186/s13059-021-02450-8 34425882 10.1186/s13059-021-02450-8 PMC8381555

[59] Kreznar JH, Keller MP, Traeger LL et al (2017) Host genotype and gut microbiome modulate insulin secretion and diet-induced metabolic phenotypes. Cell Rep 18(7):1739–1750. 10.1016/j.celrep.2017.01.062 28199845 10.1016/j.celrep.2017.01.062 PMC5325228

[60] Crèvecoeur I, Gudmundsdottir V, Vig S et al (2017) Early differences in islets from prediabetic NOD mice: combined microarray and proteomic analysis. Diabetologia 60(3):475–489. 10.1007/s00125-016-4191-1 28078386 10.1007/s00125-016-4191-1

[61] Ju Y, Janga SR, Klinngam W et al (2018) NOD and NOR mice exhibit comparable development of lacrimal gland secretory dysfunction but NOD mice have more severe autoimmune dacryoadenitis. Exp Eye Res 176:243–251. 10.1016/j.exer.2018.09.002 30201519 10.1016/j.exer.2018.09.002 PMC6215720

[62] Khokhar B, Jette N, Metcalfe A et al (2016) Systematic review of validated case definitions for diabetes in ICD-9-coded and ICD-10-coded data in adult populations. BMJ Open 6(8):e009952. 10.1136/bmjopen-2015-009952 27496226 10.1136/bmjopen-2015-009952 PMC4985868

[63] Onengut-Gumuscu S, Chen WM, Burren O et al (2015) Fine mapping of type 1 diabetes susceptibility loci and evidence for colocalization of causal variants with lymphoid gene enhancers. Nat Genet 47(4):381–386. 10.1038/ng.3245 25751624 10.1038/ng.3245 PMC4380767

[64] Oram RA, Patel K, Hill A et al (2016) A type 1 diabetes genetic risk score can aid discrimination between type 1 and type 2 diabetes in young adults. Diabetes Care 39(3):337–344. 10.2337/dc15-1111 26577414 10.2337/dc15-1111 PMC5642867

[65] Sharp SA, Rich SS, Wood AR et al (2019) Development and standardization of an improved type 1 diabetes genetic risk score for use in newborn screening and incident diagnosis. Diabetes Care 42(2):200–207. 10.2337/dc18-1785 30655379 10.2337/dc18-1785 PMC6341291

[66] Mathivanan S, Pandey A (2008) Human Proteinpedia as a resource for clinical proteomics. Mol Cell Proteomics 7(10):2038–2047. 10.1074/mcp.R800008-MCP200 18573810 10.1074/mcp.R800008-MCP200 PMC2559939

[67] Blencowe M, Furterer A, Wang Q et al (2022) IAPP-induced beta cell stress recapitulates the islet transcriptome in type 2 diabetes. Diabetologia 65:173–187. 10.1007/s00125-021-05569-2 34554282 10.1007/s00125-021-05569-2 PMC8660728

[68] Campos RK, Wong B, Xie X et al (2017) RPLP1 and RPLP2 are essential flavivirus host factors that promote early viral protein accumulation. J Virol 91(4):e01706-e01716. 10.1128/JVI.01706-16 27974556 10.1128/JVI.01706-16 PMC5286887

[69] Culton DA, Nicholas MW, Bunch DO et al (2007) Similar CD19 dysregulation in two autoantibody-associated autoimmune diseases suggests a shared mechanism of B-cell tolerance loss. J Clin Immunol 27:53–68. 10.1007/s10875-006-9051-1 17195045 10.1007/s10875-006-9051-1

[70] Gao L, Gao J, Liang Y et al (2019) Integration analysis of a miRNA-mRNA expression in A549 cells infected with a novel H3N2 swine influenza virus and the 2009 H1N1 pandemic influenza virus. Infect Genet Evol 74:103922. 10.1016/j.meegid.2019.103922 31207403 10.1016/j.meegid.2019.103922

[71] Emfinger CH, Clark LE, Yandell B et al (2023) Novel regulators of islet function identified from genetic variation in mouse islet Ca^2+^ oscillations. eLife 12:RP88189. 10.7554/eLife.88189 37787501 10.7554/eLife.88189 PMC10547476

[72] Mussmann R, Geese M, Harder F et al (2007) Inhibition of GSK3 promotes replication and survival of pancreatic beta cells. J Biol Chem 282(16):12030–12037. 10.1074/jbc.M609637200 17242403 10.1074/jbc.M609637200

[73] Hermida MA, Kumar JD, Leslie NR (2017) GSK3 and its interactions with the PI3K/AKT/mTOR signalling network. Adv Biol Regul 65:5–15. 10.1016/j.jbior.2017.06.003 28712664 10.1016/j.jbior.2017.06.003

[74] Liu T, Zhang L, Joo D, Sun SC (2017) NF-κB signaling in inflammation. Signal Transduct Target Ther 2(1):17023. 10.1038/sigtrans.2017.23 29158945 10.1038/sigtrans.2017.23 PMC5661633

[75] Darville MI, Eizirik DL (2006) Notch signaling: a mediator of β-cell de-differentiation in diabetes? Biochem Biophys Res Commun 339(4):1063–1068. 10.1016/j.bbrc.2005.11.111 16337608 10.1016/j.bbrc.2005.11.111

[76] Cain DW, Cidlowski JA (2017) Immune regulation by glucocorticoids. Nat Rev Immunol 17(4):233–247. 10.1038/nri.2017.1 28192415 10.1038/nri.2017.1 PMC9761406

[77] Luo Q, Cai Y, Zhao Q, Tian L, Liu Y, Liu WJ (2022) Effects of allopurinol on renal function in patients with diabetes: a systematic review and meta-analysis. Ren Fail 44(1):806–814. 10.1080/0886022X.2022.2068443 35856157 10.1080/0886022X.2022.2068443 PMC9307109

[78] Niu SW, Chang KT, Ta A et al (2018) Decreased incidence of diabetes in patients with gout using benzbromarone. Rheumatology 57(9):1574–1582. 10.1093/rheumatology/key138 29796661 10.1093/rheumatology/key138

[79] Beck-Engeser GB, Eilat D, Wabl M (2011) An autoimmune disease prevented by anti-retroviral drugs. Retrovirology 8:91. 10.1186/1742-4690-8-91 22067273 10.1186/1742-4690-8-91 PMC3264515

[80] Fedotcheva TA, Fedotcheva NI, Shimanovsky NL (2022) Progesterone as an anti-inflammatory drug and immunomodulator: new aspects in hormonal regulation of the inflammation. Biomolecules 12(9):1299. 10.3390/biom12091299 36139138 10.3390/biom12091299 PMC9496164

[81] Leighton E, Sainsbury CA, Jones GC (2017) A practical review of C-peptide testing in diabetes. Diabetes Ther 8:475–487. 10.1007/s13300-017-0265-4 28484968 10.1007/s13300-017-0265-4 PMC5446389

[82] Thomas HE, Kay TW (2011) Intracellular pathways of pancreatic β-cell apoptosis in type 1 diabetes. Diabetes Metab Res Rev 27(8):790–796. 10.1002/dmrr.1253 22069261 10.1002/dmrr.1253

[83] Op de Beeck A, Eizirik DL (2016) Viral infections in type 1 diabetes mellitus – why the β cells? Nat Rev Endocrinol 12(5):263–273. 10.1038/nrendo.2016.30 27020257 10.1038/nrendo.2016.30 PMC5348720

[84] Kim DY, Lee JK (2022) Type 1 and 2 diabetes are associated with reduced natural killer cell cytotoxicity. Cell Immunol 379:104578. 10.1016/j.cellimm.2022.104578 35908302 10.1016/j.cellimm.2022.104578

[85] Kracht MJ, van Lummel M, Nikolic T et al (2017) Autoimmunity against a defective ribosomal insulin gene product in type 1 diabetes. Nat Med 23(4):501–507. 10.1038/nm.4289 28263308 10.1038/nm.4289

[86] Delong T, Wiles TA, Baker RL et al (2016) Pathogenic CD4 T cells in type 1 diabetes recognize epitopes formed by peptide fusion. Science 351(6274):711–714. 10.1126/science.aad2791 26912858 10.1126/science.aad2791 PMC4884646

[87] Ratiu JJ, Racine JJ, Hasham MG et al (2017) Genetic and small molecule disruption of the AID/RAD51 axis similarly protects nonobese diabetic mice from type 1 diabetes through expansion of regulatory B lymphocytes. J Immunol 198(11):4255–4267. 10.4049/jimmunol.1700024 28461573 10.4049/jimmunol.1700024 PMC5474749

[88] Michel F, Grimaud L, Tuosto L, Acuto O (1998) Fyn and ZAP-70 are required for Vav phosphorylation in T cells stimulated by antigen-presenting cells. J Biol Chem 273(48):31932–31938. 10.1074/jbc.273.48.31932 9822663 10.1074/jbc.273.48.31932

[89] García-Bernal D, Wright N, Sotillo-Mallo E et al (2005) Vav1 and Rac control chemokine-promoted T lymphocyte adhesion mediated by the integrin α4β1. Mol Biol Cell 16(7):3223–3235. 10.1091/mbc.e04-12-1049 15872091 10.1091/mbc.E04-12-1049 PMC1165406

[90] Gautier VW, Gu L, O’Donoghue N, Pennington S, Sheehy N, Hall WW (2009) In vitro nuclear interactome of the HIV-1 Tat protein. Retrovirology 6:47. 10.1186/1742-4690-6-47 19454010 10.1186/1742-4690-6-47 PMC2702331

[91] Stanley WJ, Trivedi PM, Sutherland AP, Thomas HE, Gurzov EN (2017) Differential regulation of pro-inflammatory cytokine signalling by protein tyrosine phosphatases in pancreatic β-cells. J Mol Endocrinol 59(4):325–337. 10.1530/JME-17-0089 28827413 10.1530/JME-17-0089

[92] Kobayashi T, Toyama-Sorimachi N (2023) Metabolic control from the endolysosome: lysosome-resident amino acid transporters open novel therapeutic possibilities. Front Immunol 14:1243104. 10.3389/fimmu.2023.1243104 37781390 10.3389/fimmu.2023.1243104 PMC10540624

[93] Lincez PJ, Shanina I, Horwitz MS (2015) Reduced expression of the MDA5 gene *IFIH1* prevents autoimmune diabetes. Diabetes 64(6):2184–2193. 10.2337/db14-1223 25591872 10.2337/db14-1223

[94] Allen DW, Kim KW, Rawlinson WD, Craig ME (2018) Maternal virus infections in pregnancy and type 1 diabetes in their offspring: systematic review and meta-analysis of observational studies. Rev Med Virol 28(3):e1974. 10.1002/rmv.1974 29569297 10.1002/rmv.1974

[95] Pane JA, Fleming FE, Graham KL, Thomas HE, Kay TW, Coulson BS (2016) Rotavirus acceleration of type 1 diabetes in non-obese diabetic mice depends on type I interferon signalling. Sci Rep 6(1):29697. 10.1038/srep29697 27405244 10.1038/srep29697 PMC4942798

[96] Sixtos-Alonso MS, Sánchez-Muñoz F, Sánchez-Ávila JF et al (2011) IFN-stimulated gene expression is a useful potential molecular marker of response to antiviral treatment with Peg-IFNα 2b and ribavirin in patients with hepatitis C virus genotype 1. Arch Med Res 42(1):28–33. 10.1016/j.arcmed.2011.01.001 21376259 10.1016/j.arcmed.2011.01.001

[97] Yoshikawa A, Imagawa A, Nakata S et al (2014) Interferon stimulated gene 15 has an anti-apoptotic effect on MIN6 cells. Endocr J 61(9):883–890. 10.1507/endocrj.ej14-0219 25031023 10.1507/endocrj.ej14-0219

[98] Smyth P, Sasiwachirangkul J, Williams R, Scott CJ (2022) Cathepsin S (CTSS) activity in health and disease – a treasure trove of untapped clinical potential. Mol Aspects Med 88:101106. 10.1016/j.mam.2022.101106 35868042 10.1016/j.mam.2022.101106

[99] Noris M, Remuzzi G (2013) Overview of complement activation and regulation. Semin Nephrol 33(6):479–492. 10.1016/j.semnephrol.2013.08.001 24161035 10.1016/j.semnephrol.2013.08.001 PMC3820029

[100] Thomaidou S, Munoz Garcia A, de Lange S et al (2023) IFNɣ but not IFNα increases recognition of insulin defective ribosomal product-derived antigen to amplify islet autoimmunity. Diabetologia 66(11):2075–2086. 10.1007/s00125-023-05991-8 37581620 10.1007/s00125-023-05991-8 PMC10542729

