# Supplementary material for: Multi-tissue multi-omics integration reveals tissue-specific pathways, gene networks and drug candidates for type 1 diabetes
Source: Diabetologia. 2026 Apr 15;69(7):1911–34. doi: 10.1007/s00125-026-06721-6 (PMC13236778; doi:10.1007/s00125-026-06721-6)
Supplement: Supplementary file 1 — ESM (PDF 854 KB) [file 125_2026_6721_MOESM1_ESM.pdf]

ELECTRONIC SUPPLEMENTARY MATERIALS

Multi-tissue multi-omics integration reveals tissue-specific pathways, gene networks and drug candidates for type 1 diabetes

Montgomery Blencowe[1,2*], Zara Saleem[1], Ruoshui Liu[1,3], Margaret Wang[1], I-Hsin Tseng[1], Julian Wier[1], Stefan Mutter[4,5,6], Florin Vaida[7], Yi Guo[8], Niina Sandholm[4,5,6], Courtney Ackeifi[9], Daniel L Kaufman[10], Xia Yang[1,2,3*]

ESM Methods

Overview of study design

We utilized an integrative genomics approach that leverages multiple large-scale human genetic and genomic datasets to elucidate the genetic networks and regulators of Type 1 diabetes pathogenesis (**Fig. 1**). The datasets utilized included Type 1 diabetes GWAS from two independent cohorts, tissue specific eQTLs from diverse human tissues or cell types, various network models including gene coexpression networks, Bayesian gene regulatory networks and protein-protein interaction (PPI) networks, and biological pathway information (detailed in subsequent sections). To address reproducibility, we ran the integrative analysis on each GWAS study independently and then focused on the findings that were consistent between the two cohorts. For each GWAS, we mapped the single nucleotide polymorphisms (SNPs) to genes using tissue/cell-specific eQTL data with Marker Dependency Filtering (MDF) [1,2]. The use of eQTLs helped inform on the most likely genes affected by GWAS SNPs based on functional evidence. Next, we grouped the genes based on whether they belonged to the same biological pathways or showed coexpression, which indicated functional relevance, in data-driven gene coexpression networks. We then assessed which pathways or gene coexpression modules (a module contains genes that show coexpression patterns) demonstrated stronger genetic associations with Type 1 diabetes compared to randomly generated gene sets using Marker Set Enrichment Analysis (MSEA) [1,2]. MSEA employed a chi-square-like statistic and compared observed enrichment against 10,000 randomly permuted gene sets, with statistical significance defined as FDR < 5%. After carrying out the MSEA process for each T1D GWAS dataset, we subsequently used a Meta-MSEA to meta-analyze the two

independent GWAS data sets to look for shared pathways/modules that showed significant T1D associations, which we further simplified into independent "supersets" to reduce redundancy between pathways/modules. Integrating these Type 1 diabetes supersets with gene regulatory networks (Bayesian) and protein-protein interaction networks, we carried out the weighted key driver analysis (wKDA) to identify key drivers (KDs), which are central network genes whose network neighborhoods are highly enriched for genes in the T1D pathways and coexpression modules. KDs were selected based on enrichment significance at FDR < 5%. These KDs were then visualized in tissue-specific networks. Furthermore, we carried out *in silico* validation to determine if these key drivers are linked with T1D. Lastly, drug repositioning were done to predict the potential drugs for treating Type 1 diabetes using LINCS L1000 and PharmOmics. To further validate candidate drugs, we analyzed their association with endogenous C-peptide levels in real-world T1D patients before and after drug treatment.

Type 1 diabetes GWAS datasets

The summary statistics of GWAS for Type 1 diabetes was obtained from the JDRF/Wellcome Diabetes and Inflammation Laboratory, University of Oxford [3,4].

The study is comprised of 5913 Type 1 diabetes individuals of European descent [3,4], among which 3983 were genotyped using Illumina HumanHap550v3 (550k) Infinium Beadchip from the UK GRID and 1930 Type 1 diabetes individuals genotyped using Affymetrix 500K from the WTCCC. There were a total 8828 Controls, with 3999 genotyped using Illumina HumanHap550v3 (550k) Infinium Beadchip from the 1958 Birth Cohort (1958BC), 1490 genotyped using Affymetrix 500K (1958BC), 1455 genotyped using Affymetrix 500k from the UK Blood Services (UKBS), and 1884 genotyped using Affymetrix 500K from a cohort of bipolar disorders.

Inclusion criteria for the UK GRID are: Type 1 diabetes diagnosed between 6 months to 16 years of age, insulin dependent for greater than 6 months, a UK resident and self-identified as white European (average diagnosis age = 7.8 years of age, SD = 3.9, 47% female). Inclusion criteria for the WTCCC are: Type 1 diabetes diagnosed less

than 17 years of age, insulin dependent for greater than 6 months and self-identified white European (average diagnosis age = 7.2 years of age, SD = 3.8, 49% female). Control inclusion criteria for UKBS and 1958BC included being residents in the UK and self-identified white Europeans. For the Bipolar cohort, control individuals greater than 16 years old and resident in the UK and of European descent were included.

The above Type 1 diabetes and control individuals were partitioned into two independent cohorts based on matching genotyping platforms (i.e., Illumina or Affymetrix) between cases and controls. Cohort 1 (Affymetrix) was comprised of 1930 T1D patients and 4830 Controls. Cohort 2 (Illumina) was comprised of 3983 T1D patients and 3999 Controls.

SNP genotypes were imputed to ~10 million SNPs (1000 Genomes Phase III) using IMPUTE2, and routine quality controls were conducted as described in Cooper et al. [4]. Statistical association between SNPs and Type 1 diabetes was carried out using a Bayesian analysis. All statistical association p-values for all imputed SNPs that passed quality control, regardless of significance level for Type 1 diabetes association, were used in our downstream analyses.

Mapping SNPs to genes

To link GWAS SNPs to their potential target genes, tissue-specific eQTLs were used as they can provide functional insight for the role of SNPs in gene expression regulation within a given tissue. Thirteen eQTL sets were obtained from the GTEx database including subcutaneous adipose, visceral omentum adipose, blood, brain, colon, heart, liver, lymphocyte, muscle, pancreas, pituitary, spleen, and stomach [5,6]. Additionally, we obtained macrophage and monocyte eQTLs from the Cardiogenics Consortium [7], pancreatic islet eQTLs from various sources [8], and immune cell eQTLs including lymphocytes from the DICE study [9]. In addition, we incorporated beta-cell eQTLs from the InsPIRE consortium [10] to increase cell-type resolution within the islet. We started from the exon-level nominal results, derived Benjamini–Hochberg FDR across tests, and retained significant beta-cell eQTLs at FDR < 5%. We additionally queried for

T1D specific islet eQTLs, however Type 1 diabetes-stratified datasets were either unavailable or underpowered [11]. A broader spectrum of tissues was considered at this step to help objectively infer which tissues might be more informative for T1D association. GWAS was mapped to each tissue eQTL set separately to derive individual SNP-gene mapping sets reflecting tissue origins to allow assessment of tissue-specific signals.

A high degree of linkage disequilibrium (LD) was observed in the eQTL data, which may cause biases in the downstream analysis. For this reason, we removed redundant SNPs that had LD of r^2 >0.7 with a chosen SNP. Briefly, a GWAS SNP was compared against other SNPs for LD and T1D association. If the SNP was in LD of r^2 >0.7 with other SNPs, the one with the strongest T1D associations was chosen. This process was repeated until all remaining SNPs were not in LD based on the r^2 >0.7 cut-off. This LD pruning ensured that each association signal was independent, minimizing bias from highly linked variants. These non-redundant SNPs were used for downstream analyses.

Marker Set Enrichment Analysis (MSEA)

To identify coexpression modules and pathways that show evidence for genetic association with Type 1 diabetes, we applied MSEA from the Mergeomics package [1,2] on each of the GWAS cohorts separately in conjunction with the eQTL sources. MSEA employs a chi-square-like statistic with multiple quantile thresholds to assess whether a coexpression module or pathway shows enrichment of functional disease SNPs (i.e., those likely regulate gene expression as captured in eQTLs) compared to random chance. A null distribution was generated based on 10,000 randomly permuted gene sets for each coexpression module and pathway to control for gene set size and complexity biases. As detailed in Shu et al., the enrichment statistics from the permutations were used to approximate a Gaussian distribution from which enrichment p-values were determined [2]. Benjamini-Hochberg (BH) false discovery rate (FDR) was estimated across all coexpression modules and pathways tested for each GWAS. Gene sets were statistically significant if FDR < 5% in at least one SNP-gene mapping set. To evaluate gene sets across both GWAS studies, we followed up with a meta-analysis at the module/pathway level using the meta-MSEA function in Mergeomics, to retrieve robust

gene sets across both cohorts. Stouffer's Z score method was used to calculate meta p-values based on the p-values from the multiple MSEA runs. Meta-FDR was calculated using the Benjamini-Hochberg method, as described above.

Merging overlapping pathways into supersets

The curated pathways and gene coexpression modules may carry redundant information. For example, a KEGG pathway "insulin signalling" can have largely overlapping genes with a Reactome pathway "insulin receptor signalling". To reduce redundancy, we compared the significant modules and pathways associated with T1D at FDR <5% and merged the overlapping ones using a merging algorithm in Mergeomics to produce independent, non-overlapping "supersets" [1,2]. The algorithm employs an overlap ratio *r* between two gene sets A and B as $r = (r_{AB} \times r_{BA})^{0.5}$, where r_{AB} is the proportion of genes in A that are also present in B and r_{BA} is the proportion of genes in B which are also in A. The overlap ratio cut-off was set to r >= 0.33 and Fisher's exact test was used for assessing the statistical significance of gene overlap between modules/pathways. BH FDR < 5% was considered significant. Resultant supersets containing more than 500 genes were trimmed down to contain core genes shared among the overlapping gene sets. Following merging, 13 supersets were formed from overlapping gene sets, while 60 non-overlapping gene sets remained independent. Together, they comprise 73 final "supersets" used for downstream analysis. Supersets retained functional annotations based on dominant biological themes among contributing pathways or modules.

References

1. Ding J, Blencowe M, Nghiem T, et al. Mergeomics 2.0: a web server for multi-omics data integration to elucidate disease networks and predict therapeutics. *Nucleic Acids Res*. 2021;49(W1):W375-W387.
2. Shu L, Zhao Y, Kurt Z, et al. Mergeomics: multidimensional data integration to identify pathogenic perturbations to biological systems. *BMC Genomics*. 2016;17:1-16.
3. Barrett JC, Clayton DG, Concannon P, et al. Genome-wide association study and meta-analysis find that over 40 loci affect risk of type 1 diabetes. *Nat Genet*. 2009;41(6):703-707.
4. Cooper NJ, Wallace C, Burren O, Cutler A, Walker N, Todd JA. Type 1 diabetes genome-wide association analysis with imputation identifies five new risk regions. *BioRxiv*. Published online 2017:120022.
5. GTEx Consortium, Ardlie KG, Deluca DS, et al. The Genotype-Tissue Expression (GTEx) pilot analysis: multitissue gene regulation in humans. *Science*. 2015;348(6235):648-660.
6. GTEx Consortium. The GTEx Consortium atlas of genetic regulatory effects across human tissues. *Science*. 2020;369(6509):1318-1330.
7. Rotival M, Zeller T, Wild PS, et al. Integrating genome-wide genetic variations and monocyte expression data reveals trans-regulated gene modules in humans. *PLoS Genet*. 2011;7(12):e1002367.
8. Khamis A, Canouil M, Siddiq A, et al. Laser capture microdissection of human pancreatic islets reveals novel eQTLs associated with type 2 diabetes. *Mol Metab*. 2019;24:98-107.
9. Schmiedel BJ, Singh D, Madrigal A, et al. Impact of genetic polymorphisms on human immune cell gene expression. *Cell*. 2018;175(6):1701-1715.
10. Viñuela A, Varshney A, van de Bunt M, et al. Genetic variant effects on gene expression in human pancreatic islets and their implications for T2D. *Nat Commun*. 2020;11(1):4912.
11. Kaestner KH, Powers AC, Naji A, HPAP Consortium, Atkinson MA. NIH initiative to improve understanding of the pancreas, islet, and autoimmunity in type 1 diabetes: the Human Pancreas Analysis Program (HPAP). *Diabetes*. 2019;68(7):1394-1402.

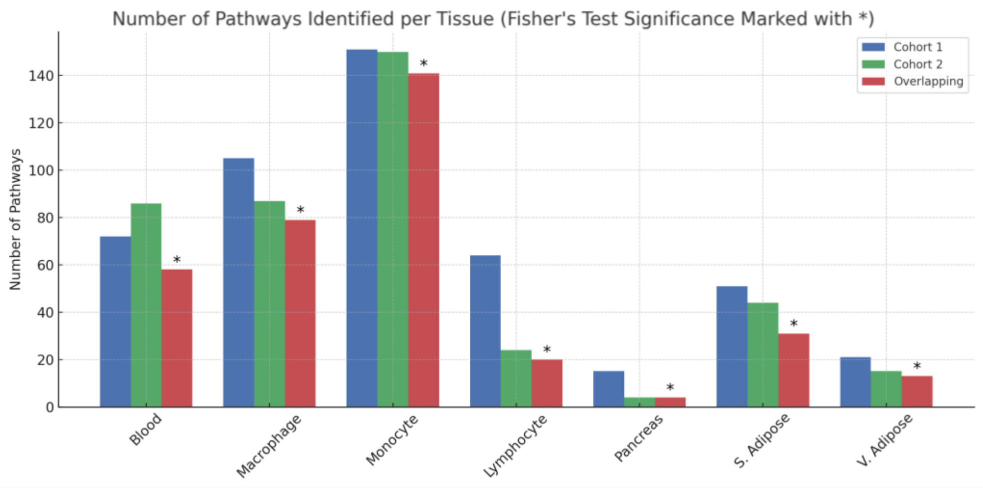

ESM Fig. 1. Number of Pathways Identified per Tissue. Fisher's Exact Test shows that all tissues demonstrated statistically significant overlap in the identified pathways between cohorts ($p < 0.05$).

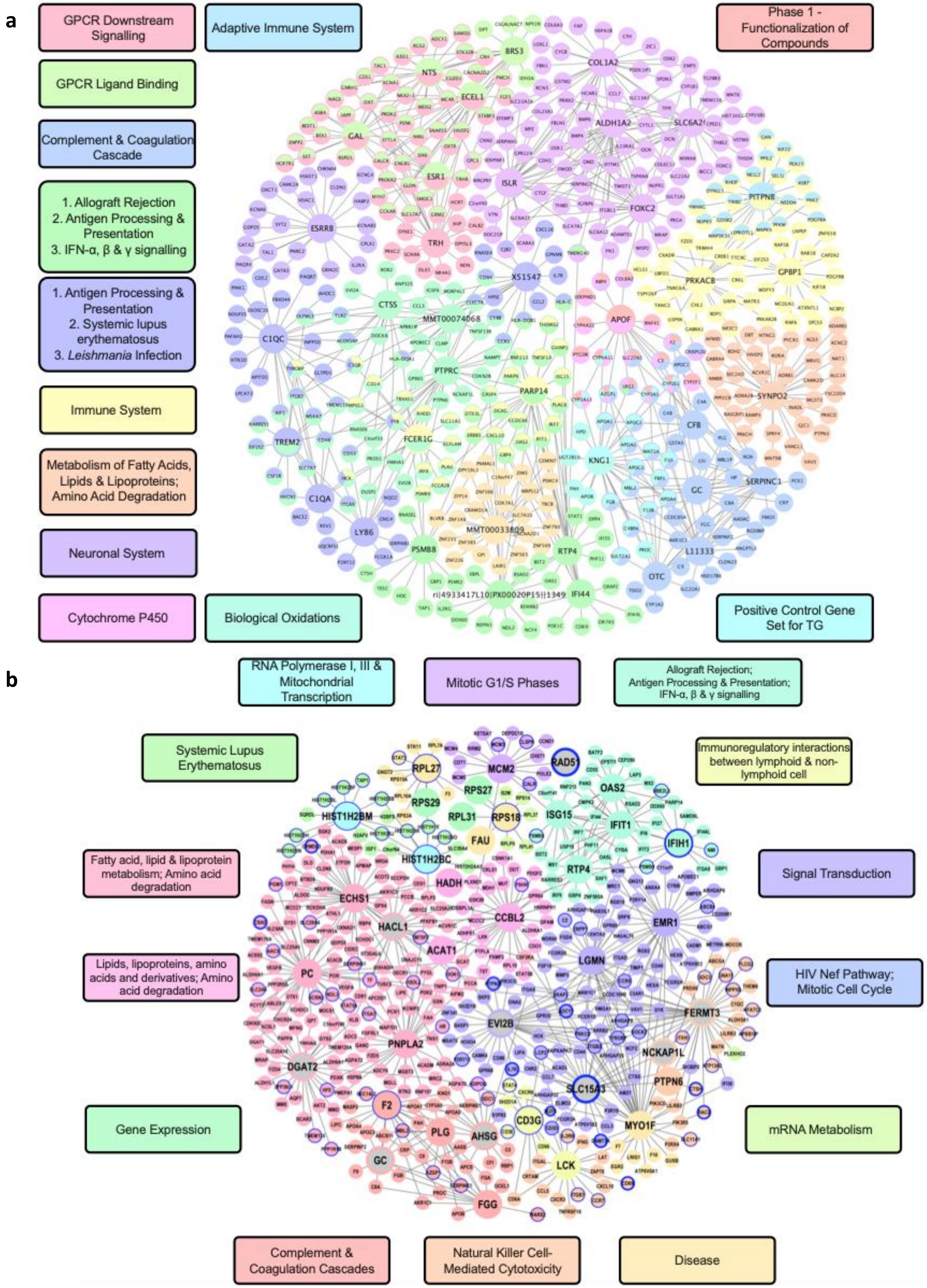

ESM Fig. 2. Bayesian Gene Regulatory Networks. (a) Islet Bayesian network. (b) Adipose Bayesian network.

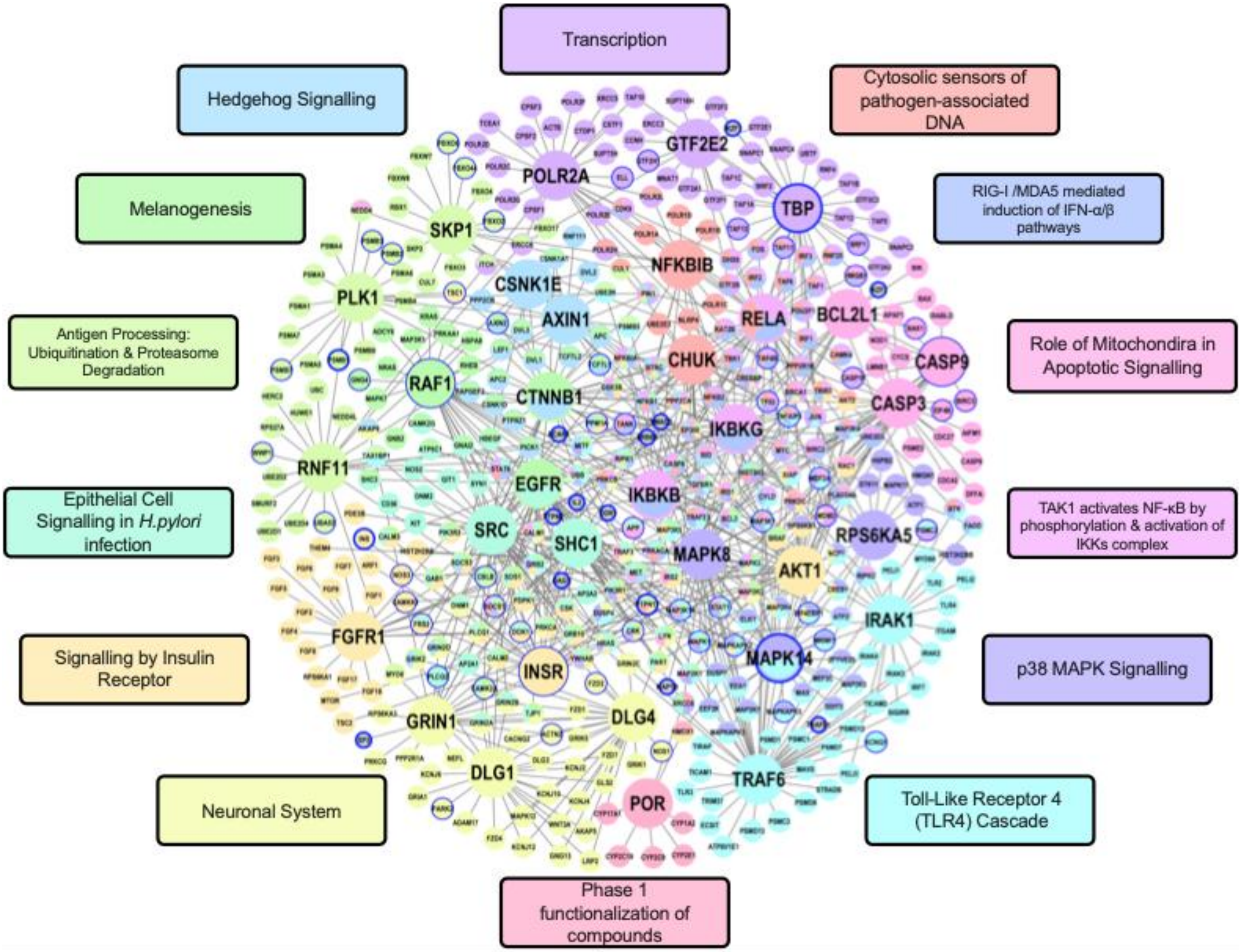

ESM Fig. 3. PPI Networks. Non-HLA Network.

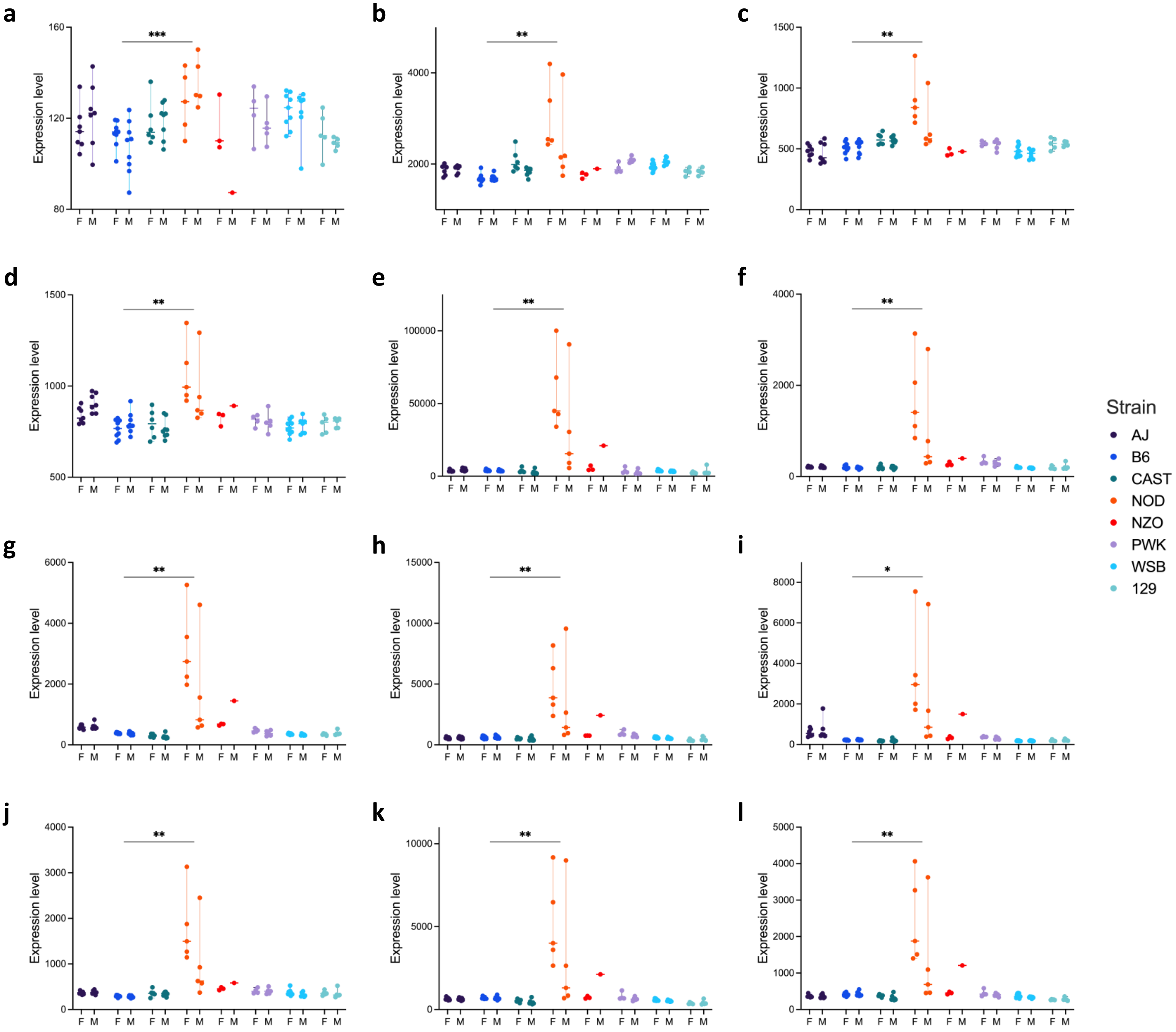

ESM Fig. 4. Example of in silico validation by screening for key driver gene RNA expression and proteomics patterns across seven non-type 1 diabetic mice with the type 1 diabetic NOD mouse. RNA expression levels of (a) *EPOR*, (b) *WASF2*, (c) *TRAF2*, (d) *SMURF1*, (e) *CD74*, (f) *NCF1*, (g) *PSMB8*, (h) *MPEG1*, (i) *GBP4*, (j) *FSCN1*, (k) *CTSS*, and (l) *C1QB* are significantly higher in NOD mouse. $p<0.05$: *; $p<0.01$: **; $p<0.001$: ***.
